# Effective Electron-Vibration Coupling by Ab Initio Methods

**DOI:** 10.1021/acs.jctc.4c01608

**Published:** 2025-02-24

**Authors:** Maximilian F. X. Dorfner, Frank Ortmann

**Affiliations:** TUM School of Natural Sciences, Technische Universität München, 85748 Garching b. München, Germany

## Abstract

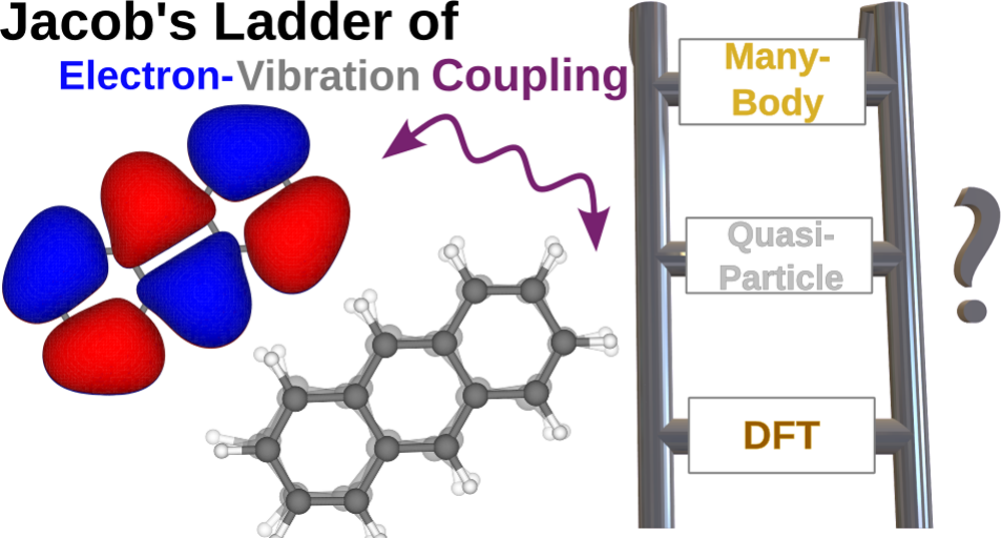

The description of electron–phonon coupling in materials is complex, with varying definitions of coupling constants in the literature and different theoretical approaches available. This article analyzes different levels of theory to introduce and compute these coupling constants. Within the quasi-particle picture, we derive an effective linear-coupling Hamiltonian, describing the interaction of electronic quasi-particles with vibrations. This description allows a comparison between coupling constants computed using density functional theory and higher-level quasi-particle approaches by identifying the Kohn–Sham potential as an approximation to the frequency-independent part of the self-energy. We also investigate their dependence on the exchange-correlation (XC) functional. Despite significant deviations of the Kohn–Sham eigenvalues, which arise from different XC functionals, the resulting coupling constants are remarkably similar. A comparison to quasi-particle methods, such as the well-established G0W0 approach, reveals significant quasi-particle weight renormalization. Surprisingly, however, in nearly all the considered cases, the coupling constants computed in the DFT framework are excellent approximates of the ones in the quasi-particle framework, which is traced back to a significant cancellation of competing terms. Other quasi-particle methods, such as the Outer Valence Green’s Function approach and the Δ*S*CF method, are also included in the comparison. Moreover, we investigate the coupling of vibrations to excitonic excitations and find, by comparison to time-dependent density functional theory and extended multiconfiguration quasi-degenerate second-order perturbation theory, that knowing the underlying electron- and hole-vibration couplings is sufficient to accurately determine the exciton-vibration coupling constants in the studied cases.

## 1 Introduction

Many phenomena in physical chemistry and condensed matter physics are due to the dynamic interplay of nuclear and electronic motions, eventually resulting from the interaction between these degrees of freedom. This includes, for example, conventional superconductivity^1−4^ or metal–insulator transitions,^5−7^ where the coupled motion of electrons and nuclei ultimately leads to a different phase of matter, which alters the material’s properties significantly. Beyond these almost iconic examples, numerous material properties are strongly influenced by this interaction, including technologically relevant aspects such as heat transport,^8−10^ energy transfer,^11−13^ charge-carrier transport,^14−19^ the optical response of materials,^20,21^ or efficiencies of solar cells.^22−24^

These examples emphasize the relevance of electron-vibration couplings for applications and highlight the necessity of a combined treatment of electronic and nuclear motion in theoretical approaches. Unfortunately, treating the nuclei positions entirely as quantum variables introduces overwhelming complexity.

**Figure 1:**
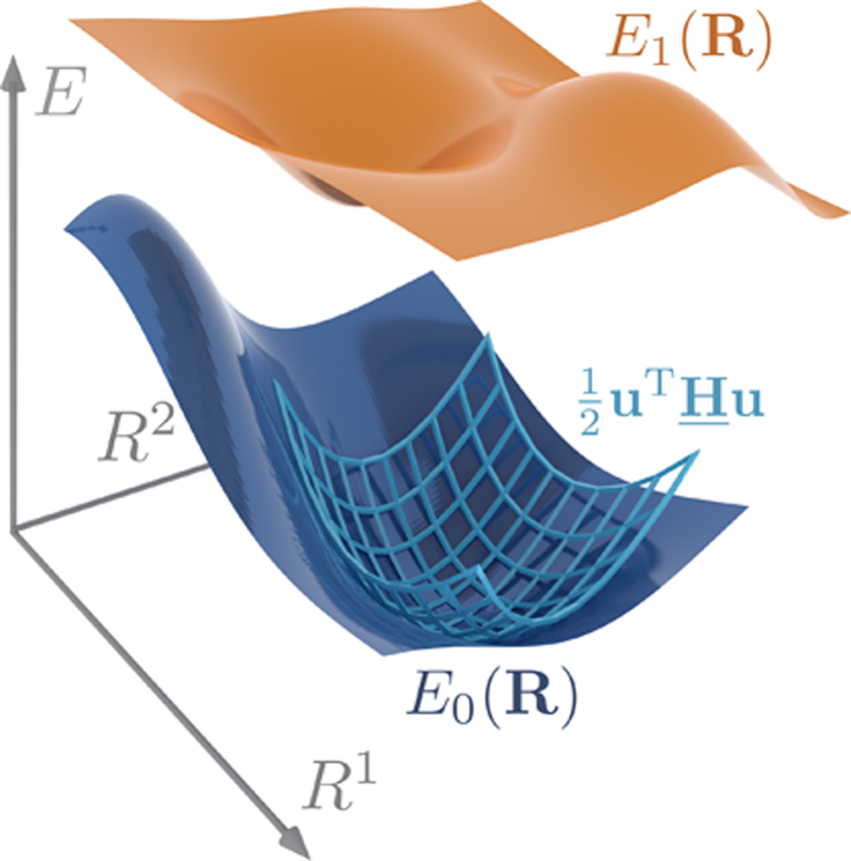
Schematic illustration of two potential energy surfaces, with quadratic approximation at the minimum nuclear geometry **R**^0^ of the ground-state PES.

A first simpler level of description, therefore, treats the nuclei as quasi-classical objects and is based on the Born–Oppenheimer (BO) decoupling,^25^ wherein the motion of the nuclei is first neglected and the concept of potential energy surfaces (PES) shown in Figure 1 is introduced. To go beyond fixed atoms, the nuclear kinetic energy is reintroduced, such as in conventional ab initio molecular dynamics^26−31^ where a simplified version of the electron-nuclei interaction is included. Alternatively, a quantum description of the nuclei can be pursued. A minimal but approximate way of doing this is by assuming separable electrons and vibrations (phonons). Then, the nuclear kinetic energy operator  is diagonal in the electronic states. This approximation still neglects the interstate coupling, e.g., between the states *E*_0_ and *E*_1_ in Figure 1, by . This might be permissible only for large energy differences between the states, such as in small molecules in gas phase. However, in many real-world situations, a small level separation is encountered, such as near degeneracies of PES at conical intersections, in aggregated systems, semiconductors, and other bulk materials.^32^ In these cases, the neglect of interstate coupling becomes invalid. Therefore, the quantum chemistry community has introduced the linear vibronic coupling model, which includes interstate coupling.^32−34^

The approach to obtain these coupling constants, starting from the BO decoupled Hamiltonian , is finding an energetic minimum **R**_0_ of the ground state PES *E*_0_(**R**) [represented by the parabola in Figure 1], calculating normal modes and computing the matrix elements of the operator  for many-body electronic states and their projection onto the normal modes. While this full many-body approach is conceptually clear, it remains equally impractical because the purely electronic problem is already nearly unsolvable for all but the simplest systems. The most accurate approximations of this ideal scenario are achieved through various high-level quantum chemistry methods that have been applied in the past.^32,35−40^ These methods, in some or the other way go beyond single-particle approximations of the full many-body Hamiltonian, and we collectively refer to them as many-body approaches. Although desirable, these ab initio many-body approaches are unfeasible for real materials, ranging from large molecules and extended molecular structures to periodic or even disordered condensed-matter systems. Only with the advent of density functional theory (DFT)^41,42^ the ab initio study of the electron–phonon coupling (EPC) in realistic solid-state systems became possible and routinely used. In this case, the Born–Oppenheimer decoupled Hamiltonian is replaced by the Kohn–Sham matrix, which is the standard approach for calculating the EPC constants in these systems today.^43^

At first glance, DFT and many-body approaches to obtain EPC constants might appear similar when the many-body states and energies are replaced by Kohn–Sham orbitals and eigenvalues in DFT. However, this similarity is misleading, potentially leading to incorrect interpretations or conclusions. Strictly speaking, the Kohn–Sham orbitals and their corresponding eigenvalues have no rigorous mathematical relation to the many-body states and energies, as they constitute only an auxiliary system to represent the electron density.^42^ As a consequence, the standard DFT approach to calculate EPC constants lacks a formal theoretical foundation,^43^ although it is commonly used. It is, therefore, a great dilemma that, up to now, there is no clear theoretical connection between the coupling constants computed using the DFT approach and the ones from an original many-body approach.

To clarify this connection, we investigate different levels of theory to compute the linear EPC. We discuss an ab initio linear coupling quasi-particle Hamiltonian and conditions for its validity as a common conceptual framework for several closely related approximation schemes. We discuss conditions under which DFT coupling constants are good approximations to those determined through a quasi-particle method, like the Outer Valence Green’s function theory,^44^ or the commonly used G0W0^45,46^ approach. Toward this goal, we begin with a concise yet essential introduction to the quasi-particle concept, the framework for our analysis, and the linear coupling constants we derive. In addition to this analytical analysis, we test our considerations numerically for several molecules. Although quasi-particle approaches for calculating EPC parameters are naturally limited to systems of the size of such molecules, the analyzed systems consistently demonstrate agreement in the coupling constants across various theoretical frameworks.

## 2 Effective Electronic Structure Approaches

### 2.1 Initial and Effective Hamiltonians

In this work, we consider interacting electrons in an external potential from the nuclei, whose positions and momenta are themselves regarded as dynamical quantum degrees of freedom. The Hamiltonian describing these situations is given by
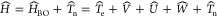
1where *T̂*_e_ is the kinetic energy of the electrons, *V̂* the external potential provided by the nuclei, *Û* is the Coulomb interaction between the electrons, *T̂*_n_ is the kinetic energy of the nuclei and *Ŵ* is the interaction of the nuclei among each other. We restrict ourselves to systems with a unique, sufficiently gapped, spin-singlet ground state. When choosing an orthonormal single-particle basis {|*x*⟩} for the electronic degrees of freedom, the (Born–Oppenheimer) Hamiltonian can be written in the form
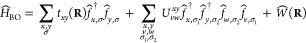
2Here  and  denote Fermionic creation and annihilation operators in orbital |*x*⟩ (with ϕ_*x*_(**r**) = ⟨**r**|*x*⟩) and spin projection σ. **R** denotes the set of all nuclear position operators. The matrix elements *t*_*xy*_(**R**) are **R**-dependent through the external potential
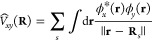
3where **R**_*s*_ is the position of the nucleus *s* and atomic units are employed. Furthermore,  denote the Coulomb matrix elements in the chosen orbital basis, which, by our choice, do not depend on the nuclear coordinates **R**. The Coulomb interaction  usually requires explicitly considering a Hilbert space that scales exponentially with the number of electrons *N* to find the eigenstates |α⟩_*N*_ of , with energy . As this is prohibitive for bulk systems, it is necessary to replace the formally exact description based on  by an approximate one under the constraint that the low-energy excitations (above some reference ground state |0⟩_*N*_) are approximately reproduced. This can be achieved by replacing  with an effective Hamiltonian of the form
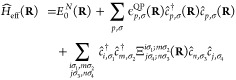
4Here  are Fermionic operators that create and annihilate electrons in orthogonal orbitals labeled by {*p*} = {*i*}∪{*m*}, where we index electron excitations by *i* and hole excitations by *m*, on top of the given ground state |0⟩_*N*_. This means one assumes that  = 0 holds for electron-type (de)-excitations and analogously for holes. In both cases, σ denotes the spin index. Note that the form of eq 4 restricts the following discussions to systems that conserve the spin-projection. Furthermore,  are the energies of these states, and  denote the matrix elements of the effective electron–hole interaction. An important difference to the bare Coulomb interaction  in eq 1 is that Ξ contains the screening that is produced by all the electrons present in |0⟩_*N*_. The **R**-dependence signifies the overall parametric dependence on the nuclear coordinates. Clearly, the form of the Hamiltonian (eq 4) can be extended beyond electron–hole interaction to further include electron–electron and hole–hole interactions as well as higher-order terms in the interaction between electronic excitations above |0⟩_*N*_, which, however, is not necessary for our purposes here. Our approach here is similar to ref (47).

### 2.2 Dyson Orbitals and Effective Interaction

A natural way to obtain an effective Hamiltonian in the form of eq 4 is to employ a Dyson orbital description.^48−50^ To simplify the notation, we will omit the dependence on nuclear coordinates in this subsection. The electron Dyson spin orbitals ψ_σ_^(α)^(**r**, *N* + 1) are in general defined by^51,52^
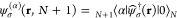
5where |α⟩_*N*+1_ denotes the α^th^ eigenstate of the electronically fully interacting Hamiltonian eq 2 with *N* + 1 electrons and  denote the electronic field creation operator. The Dyson orbitals therefore describe the amplitudes associated with adding an electron to |0⟩_*N*_. One can associate a single-particle state |*i*, σ⟩ with this Dyson orbital, via the identification
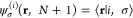
6Analogously one can associate hole states {|*m*, σ⟩} with the hole Dyson orbitals. However, one should keep in mind that these states form an overcomplete, unnormalized, and nonorthogonal basis.

As long as the spectrum of single-particle excitations is discrete one can associate the states |*p*, σ⟩ with the energies
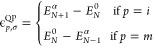
7which follows from the poles of the single-particle Green’s function in the Lehmann representation. Alternatively to the (impractical) projection of the exact many-body eigenstates in eq 5, the Dyson orbitals and their energies can be obtained from the quasi-particle equation^51,53^
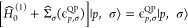
8where the self-energy operator (ω), derived from the Dyson equation for the single-particle Green’s function, constitutes a nonlocal, nonhermitian, energy-dependent effective potential generated by the interaction between the particles.  denotes the sum of a single electron’s kinetic energy and the nuclei’s external potential.

To include also neutral excitations into the effective theory, an effective, frequency-dependent, two-particle (electron–hole pair) Hamiltonian can be derived,^54,55^ consisting of the kinetic energy, the external potential and self-energies for quasi-electron and quasi-hole, as well as the two-particle version of the self-energy (ω), which describes the effective interaction between quasi-electron and quasi-hole. Analogous to the quasi-particle equation eq 8, the exciton quasi-particle states |*A*_*k*_⟩ obey the equation
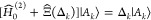
9in which  are the excitation energies of the exact singlet states. In both cases represented by eqs 8 and 9, the excitation energies are contained in the self-energy operators, and hence, self-consistent solutions have to be found. The corresponding eigenstates belonging to different energies are, in general, nonorthogonal. While this nonorthogonality can, in principle, be accounted for by introducing the corresponding overlap matrix (or its inverse), it is not considered here, as the states are, in many cases, nearly orthogonal.

### 2.3 Quasi-Particle Approximations

These effective one- or two-particle theories are, in principle, exact effective theories but have several technical difficulties, which complicate their practical application, not only for the inclusion of electron–phonon interaction but also for finding the Dyson orbitals and their energies. Most of the complications are rooted in the nonhermiticity of the effective Hamiltonians eqs 8 and 9. Although, there are approaches that avoid this nonhermiticity by construction, such as some versions of coupled cluster theory^56^ or the algebraic diagrammatic construction scheme,^57,58^ quite commonly, the antihermitian part of (ω) or (ω) is just neglected.^55,59^ We collect these type of approximations that eliminate this nonhermiticity under the name of quasi-particle approximations. Specifically, the exact self-energy (and any approximation thereof) can be decomposed into a frequency-independent, hermitian part  and a residual, frequency-dependent contribution (ω)^50,60^ of the form
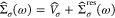
10where the residual self-energy (ω) is a causal function. The specific flavor of quasi-particle approximation that we consider here assumes that the self-energy is diagonal in the eigenstates of , as otherwise, the eigenstates are energy-dependent and thus, in general, nonorthogonal, and that there is a negligible imaginary part. Formally, this can be written as,
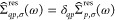
11where |*p*, σ⟩ and |*q*, σ⟩ are exact eigenstates of , and . {|*p*, σ⟩} is then the basis to represent eq 4. Similarly, for neutral excitations, one may obtain a hermitian effective interaction by employing a static approximation and the Tamm-Dancoff approximation, as, e.g., discussed in refs (53) and (61−63) Several flavors of approximations are considered in this work and will be discussed at the time of their occurrence.

In cases where the hermitian terms  dominate over (ω), one can expect that the Dyson orbitals are close to {|*p*, σ⟩}, such that the effective Hamiltonian in eq 4 will be a good replacement to describe excitations. While in principle, the above construction to obtain the effective parameters entering eq 4 can be pursued in any case, its quality to approximate the initial Hamiltonian eq 1 is tied to the smallness of the imaginary part of the self-energy near the quasi-particle energies, i.e., , compared to . This is because the former determines the lifetime of the quasi-particle states, which should be longer than other relevant time scales for the model to be suitable.

In the present context, it should be emphasized that this criterion for quasi-particles can be studied for any given nuclear arrangement **R**_0_. If this is valid, still, any other geometry **R** must be reassessed. Phrased differently, only because the system supports quasi-particles at the reference geometry **R**^0^, one cannot infer their existence at a different **R**. For what follows, it is necessary that the quasi-particle states remain stable in the proximity of the reference geometry. Stability in this context means that if a certain system supports quasi-particles at the ground state geometry **R**^0^, it should also support quasi-particles at a slightly displaced geometry **R**^0^ + **u**. As this is encoded in the imaginary part of the self-energy and its derivative with respect to the nuclear geometry **R**, we assume a vanishing derivative for the rest of the article because instabilities of quasi-particles are not the focus of this work.

### 2.4 Effective Second-Quantized Linear-Coupling Hamiltonian

We consider cases in which  has a minimum of the potential energy surface at nuclear configuration **R**^0^ and expand all terms in eq 4 that depend on **R**, in the displacements **u** from this minimum configuration **R** = **R**^0^ + **u**. At the minimum **R**^0^, we transform from nuclear coordinates into normal modes and their displacement operators *X*^λ^. These are expressed in terms of the bosonic creation and annihilation operators  and , respectively, for each mode λ. Full details are summarized in the Supporting Information Section S1. Collecting all terms up to the first nonvanishing order, one finds
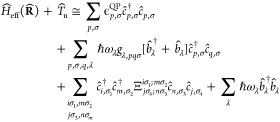
12where the abbreviations  are used for the electron annihilation and creation operators at the reference geometry. *ℏ*ω_λ_ is the energy quanta of the harmonic oscillator λ.  are the linear coupling constants given by
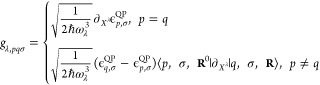
13Note that in this work, when referring to coupling constants, we mean the dimensionless linear electron–phonon coupling constants. To further unify the nomenclature in the following, let us call (*p* = *q*) the diagonal coupling constants, as these only involve the diagonal eigenenergies. Consequently, we call (*p* ≠ *q*) the off-diagonal coupling constants throughout.

Furthermore,  in eq 12 are the interaction matrix elements at the reference geometry in a frozen orbital basis. At this point, we note that the electronic screening may ultimately cause a residual **R** dependence of the matrix elements  even in an **R**-independent (frozen-orbital) basis and Ξ could still change with nuclei displacements. However, we neglect this dependence for the gapped systems we address here, as this should be rather small. That is, screening should be weakly affected by slight changes in the geometry, at least in cases of stable quasi-particles for gapped systems considered here; hence, ∂_**R**_Ξ ≈ 0. This approximation has been numerically tested within the GW-BSE level of theory in ref (64) and within GW and COHSEX level of theory in ref (65). Therein, it was found that this approximation is “excellent in practice”. This is supported by our analytic analysis of the screening upon nuclei displacement in the Supporting Information Section S2. As a result, we conclude that the coupling of the vibrations to the electronic degrees of freedom in eq 12 is dominated by the **R** dependence of the quasi-particle states and energies. The implications of this point should be sufficiently emphasized, namely that in this approach, the linear quasi-particle coupling constants  fully determine the linear excitonic coupling constants. This will be discussed in more depth later in this article.

## 3 From Quasi-Particles to a Practical Scheme

While the considerations in previous sections are crucial for understanding the physics and possible limitations of a quasi-particle-based approach to EPC, one must select a specific version of quasi-particle theory to parametrize the effective Hamiltonian eq 12. For extended systems, a quasi-particle approach based on the exact self-energy is of limited practical use for coupling constants. This is because, although single quasi-particle calculations are feasible even for large molecules or crystalline structures,^66,67^ the number of calculations required to determine the coupling constants based on a frequency-dependent self-energy is typically prohibitively large, making approximations necessary. Frequently, as an approximation to compute coupling constants, the self-energy in eq 8 is replaced by the Kohn–Sham potential,^15,68−72^ which we refer to as Kohn–Sham (KS) approximation to the quasi-particle coupling constants. This approach represents a natural extension of the common practice of using the KS potential  to approximate the self-energy (ω)^62,63^ as a starting point for perturbative treatments.^73,74^

Before examining the accuracy of this approximation and identifying the conditions under which it can fail, it is important to note that the exact Kohn–Sham (KS) potential is not available in practice. Instead, one relies on various approximate exchange-correlation (XC) functionals, raising the question of how strongly the KS coupling constants depend on the chosen XC functional, which we address first. Our numerical results in the following section have all been obtained based on the CP2K software package,^75^ along with our own extensions for computing coupling constants.

### 3.1 Functional Dependence of the KS Coupling Constants

We investigate the overall consistency of the KS coupling constants obtained by different functionals, including LDA, PBE,^76^ PBE0,^77,78^ BLYP,^79,80^ B3LYP^79−81^ and CAM-B3LYP.^82^ To address this question for specific cases, we study the pyrazine molecule, which is a prototypical example for strong diagonal- and off-diagonal coupling, both for charged^83^ and charge neutral excitations.^35,84^

We perform geometry optimizations of the neutral molecule using cc-TZV2P basis sets from the HFX basis set collection in CP2K and GTH pseudopotentials^85,86^ with the corresponding XC functional. Afterward, the normal modes and normal mode energies are computed using the numerical diagonalization of the Hessian. Some vibrational modes do not couple significantly with the electronic states and are left out of the comparison. We identify the relevant normal modes of pyrazine with {*v*_6*a*_, *v*_1_, *v*_9*a*_, *v*_8*a*_, *v*_2_, *v*_10*a*_} (using the nomenclature from ref (35)) by symmetry, closeness in energy, and by their normal mode pattern. The KS state-diagonal coupling constants of the two lowest hole states, denoted by *m* = 0 and *m* = −1, are obtained as discretized derivatives of the KS eigenvalues along the normal modes. The off-diagonal coupling matrix elements that are induced by the mode *v*_10*a*_ are computed as
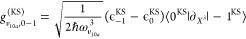
14where the subscripts 0 and −1 label the HOMO and HOMO–1 orbitals, respectively. Note that for an atom-centered basis (as used in CP2K), the calculation of the derivatives also requires a basis transformation from the displaced basis to the basis used at the equilibrium geometry, which we implemented to get accurate results. Further technical details and a convergence check of the KS coupling constants with respect to the used basis set size and subsequent discussion can be found in Supporting Information Sections S3.1 and S3.3.

**Figure 2:**
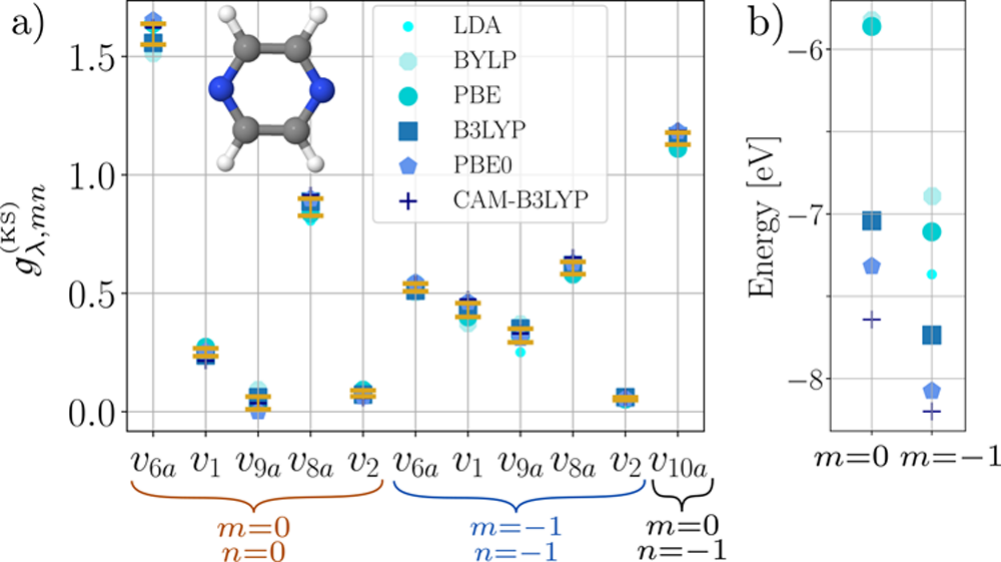
Hole coupling constants of pyrazine. (a) Comparison of the lowest two hole states *m* ∈ {0, – 1} for the totally symmetric modes {*v*_6*a*_, *v*_1_, *v*_9*a*_, *v*_8*a*_, *v*_2_} and the mode *v*_10*a*_ computed by using different XC functionals. Candlesticks indicate the MAD with respect to the mean of all functionals. (b) Kohn–Sham eigenvalues of hole states *m* ∈ {−1, 0}. Legend applies to both subfigures.

Figure 2a summarizes the coupling constants for the relevant modes and the considered (occupied) states. The values vary only slightly among the used functionals for each mode and state. This is quite remarkable when considering the spread of the Kohn–Sham eigenvalues for *m* = −1, 0 for these functionals in Figure 2b. While the eigenvalues deviate by up to 1.5 eV, the coupling constants in (a) agree very well. We quantify this remarkable agreement by computing the mean absolute difference (MAD), depicted by the error bars for each combination of mode and state. The mean of all the MAD values (MMAD) over all the various studied modes for a fixed state reflects the overall variance of the coupling constants across different functionals with respect to the mean. It is calculated to be 0.010 for the HOMO (state pair *m* = *n* = 0) and to 0.008 for the HOMO–1 (*m* = *n* = −1). These very low values lead us to conclude that, although we considered vastly different XC functionals, the respective derivatives and, thus, the diagonal coupling constants are remarkably insensitive to changing the functional. The same applies to the relevant off-diagonal coupling constant (*m* = 0, *n* = −1) with MMAD = 0.025, which we consider here. As discussed in the Supporting Information Sections S3.2.3 and S3.2.4 to this article, these values are also close to those from the Outer Valence Green’s function result from ref (83). The above findings suggest restricting further numerical comparisons to a single XC functional in the following sections.

### 3.2 Analytical and Numerical Analysis: QP vs KS Coupling Constants

We now analyze the relation between the coupling constants in the KS description  and the QP coupling constants. For this, it is assumed in this section that we have a reference potential  at hand, such that |*p*⟩ = |*p*^KS^⟩ and the ground state is a spin-singlet, such that we can drop any spin-labels.

#### 3.2.1 Diagonal Elements of the Coupling Matrix

The main difference between quasi-particle states and states originating from a hermitian potential, like the Kohn–Sham potential, is the finite lifetime and the reduced weight in their spectral functions, described by a renormalization factor *Z_p_* < 1. This quasi-particle renormalization may naturally appear when the inner product of the left and right eigenstates of the effective Hamiltonian are concerned. As the electron–phonon coupling matrix elements are similarly defined, one can hypothesize that this renormalization should naturally carry over to the electron–phonon coupling. This could motivate the ad hoc ansatz to renormalize the KS coupling constants by the quasi-particle weight *Z_p_*. As a result, this would give systematically lower QP coupling values. However, the situation is more complex, and this ad hoc ansatz is not permitted.

To show this, we rewrite the diagonal coupling constants (*p* = *q*) in eq 13, with the help of eq 8 and the Feynman-Hellmann theorem, as
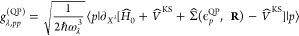
15By separating the first two terms from the last two terms and replacing the exact quasi-particle states with the eigenstates of the Kohn–Sham system |*p*⟩ →|*p*^KS^⟩ in the first two terms, one finds with the help of condition eq 11, the relation
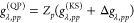
16where  are the coupling constants in the Kohn–Sham reference system,  is the quasi-particle weight of the quasi-particle state and . Here we further made use of the shorthand notation δΣ̂(ω, **R**) for the difference of the Kohn–Sham potential and the self-energy  = .

In this result, the hypothesized renormalization *Z*_*p*_ between KS and QP coupling constants indeed occurs but is modified by another term Δ*g*_λ,*pp*_ equally multiplied by the quasi-particle weight. The appearance of different terms in eq 16 results from the self-consistency condition in the quasi-particle equation eq 8. *Z*_*p*_ incorporates the change in the nuclear geometry δ**R** only implicitly, i.e., through the self-consistently determined quasi-particle energy. In contrast, the correction term Δ*g*_λ,*pp*_ in eq 16 measures the explicit nuclear dependence of the self-energy beyond the Kohn–Sham potential that does not enter through the quasi-particle energy.

To shed more light on the relation between KS and QP coupling constants and the different contributions, we test eq 16 numerically. Toward this end, we consider the anthracene molecule and compute the ground state equilibrium geometry of the neutral molecule and its normal modes with the CAM-B3LYP hybrid functional.^82^ We employ the cc-TZV2P basis sets from the HFX-basis set collection in CP2K^75^ and GTH pseudopotentials.^85,86^ The G0W0 method is chosen as the specific flavor of the quasi-particle method for the comparison. We employ the resolution-of-identity approach of refs (87) and (88) as implemented in CP2K. Our analysis focuses on the totally symmetric mode with energy . We compute the electron and hole coupling constants  by a finite difference approach to ∂_*X*^λ^_ϵ_*p*_^(G0W0)^. Similarly, the coupling constants in the Kohn–Sham framework are computed as discretized derivatives of the KS eigenvalues. The Δ*g*_λ,*pp*_ is obtained as finite-difference derivative ∂_*X*^ν^_[Σ_*p*,σ_(ω, **R**) – *V*_*pp*_^KS^(**R**)] for every (fixed) ω. Finally, we evaluate the frequency variable at the respective quasi-particle energy at the equilibrium geometry. Table 1 compares the individual contributions in eq 16 to the G0W0 result for the ten lowest electron and hole states. From the difference in the last column, we find that the equation is numerically almost perfectly fulfilled, confirming our analytical result.

**Table 1:** Comparison of the Anthracene Molecule’s Diagonal Coupling Constants for the Considered Mode ν for the Ten Lowest Electron and Hole Excited States^a^

*p*	*Z*_*p*_					δ (eq 16)
–9	0.814	0.5854	0.5707	0.1491	0.5857	0.0003
–8	0.745	0.0988	0.0129	0.1198	0.0989	0.0001
–7	0.833	–0.7083	–0.7026	–0.1473	–0.7082	0.0001
–6	0.842	0.8237	0.8154	0.1626	0.8235	0.0002
–5	0.848	–0.8694	–0.8701	–0.1549	–0.8691	0.0003
–4	0.762	0.1301	0.0712	0.0994	0.1299	0.0002
–3	0.819	0.3572	0.3694	0.0667	0.3572	0.0000
–2	0.830	0.3572	0.3626	0.0675	0.3570	0.0002
–1	0.861	–0.3676	–0.3817	–0.0450	–0.3675	0.0001
0	0.865	0.4496	0.4435	0.0763	0.4496	0.0000
1	0.879	–0.3285	–0.3206	–0.0530	–0.3283	0.0001
2	0.883	0.4658	0.4607	0.0670	0.4659	0.0001
3	0.867	–0.4320	–0.4313	–0.0669	–0.4317	0.0003
4	0.886	–0.5689	–0.5524	–0.0898	–0.5687	0.0002
5	0.946	0.0083	0.0212	–0.0124	0.0083	0.0000
6	0.940	0.0029	0.0119	–0.0085	0.0032	0.0003
7	0.936	–0.0255	–0.0226	–0.0045	–0.0254	0.0001
8	0.945	–0.0410	–0.0316	–0.0121	–0.0413	0.0003
9	0.941	–0.0058	–0.0126	0.0067	–0.0055	0.0003
10	0.826	–0.0295	–0.0029	–0.0327	–0.0293	0.0002

a Displayed (from left to right) are the quasi-particle weight, the quasi-particle coupling constants (finite difference approach), the Kohn-Sham coupling constants, the correction term, and the r.h.s. of eq 16, which should match the quasi-particle coupling constants. The last column shows the absolute value of the difference of the left and r.h.s of eq 16 as a measure for consistency.

This agreement of the analytical and numerical results allows us to study the contribution of the individual components (*Z*_*p*_,  and ). By comparing the values of  and  in Table 1, we find that in almost all the considered cases (except *p* = −8, – 4, 10), the Kohn–Sham coupling constant is the dominant contribution to the quasi-particle coupling constant. It is, however, significantly renormalized by *Z*_*p*_, which amounts to 0.863 on average. Nonetheless, contrary to our previous belief, the correction term  is, in many cases, sizable.

On the other hand, if we disregard the analytical relationship between  and  and simply compared the obtained values, we find that the latter is, in many cases, an excellent approximation to the former, suggesting that the Kohn–Sham approximation
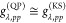
17works very well. This is particularly intriguing as it would suggest that the KS level of theory is sufficient for calculating coupling constants at the G0W0 level, thus leading to great savings in computational resources.

We can also identify the reason for this: Although the correction term  can be quite sizable, when simultaneously neglecting the quasi-particle weight factor *Z*_*p*_ and this correction term, the resulting error is much smaller, compared to a neglect of one or the other. This suggests that a significant error cancellation makes eq 17 good in practice.

Still, there are cases where eq 17 fails. For example, for *p* = −8 or *p* = −4, where the correction term is the largest contribution, the replacement of  by  would lead to a strong underestimation of the G0W0 coupling constants by the Kohn–Sham approximation. As the quasi-particle weight is the smallest for these cases, these deviations suggests a relation to the quality of the quasi-particle description. Contrary to that, in the case *p* = 5, the quasi-particle weight is close to unity, still deviations are relatively large. We can also observe the reason for this, as  has a different sign than , making the error cancellation impossible. Nonetheless, all these deviations are bearable, as the affected coupling constants are relatively small anyway, such that the physics that is usually dominated by the larger couplings remains the same.

#### 3.2.2 Off-Diagonal Elements of the Coupling Matrix

Focusing now on the off-diagonal coupling (*p* ≠ *q*), we first note that, under the made assumptions, we can write , which allows decomposing the off-diagonal coupling constants in eq 13, as
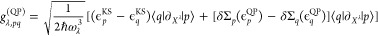
18By replacing the exact quasi-particle states with the eigenstates of the Kohn–Sham system |*p*⟩ → |*p*^KS^⟩, we arrive at
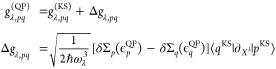
19with the Kohn–Sham coupling constants . Notably, the corrections to the bare Kohn–Sham coupling constants by Δ*g*_λ,*pq*_ do not include a quasi-particle weight factor. This is a difference to the diagonal case and a consequence of the orthogonality condition eq 11.

We first test the analytical result, eq 19, for anthracene by comparing the G0W0 coupling constants to alternative ones obtained without using this equation (cf. Supporting Information, S3.4.2). The alternative approach that serves as a benchmark here uses the G0W0 potential energy surface and employs a diabatization procedure (DP) using only G0W0 energies along the displaced normal modes. As this depends on the feasibility of the DP, we have to restrict ourselves to two neighboring states, namely HOMO and HOMO–1 and a strongly coupling mode. A detailed discussion of the DP is summarized in Section S3.4.2. We find the corresponding DP coupling constant *g*_–1, 0_^G0W0-DP^ = 0.486 to be close to the one obtained from eq 19 (*g*_–1, 0_^G0W0^ = 0.469). We, therefore, continue with the approach in eq 19.

The off-diagonal correction Δ*g*_λ,*pq*_ to the KS coupling constants is of interest. According to eq 19, it scales with the difference of the quasi-particle shifts δΣ(ω) taken at both involved state energies. Quasi-particle shifts are known to be different for electrons and holes, which leads to the well-known gap correction and may directly impact the coupling constants via eq 19. Thus, from the perspective of possibly simplifying the calculations of  by just using , this result appears disappointing, because with underestimated Kohn–Sham DFT energy gaps,^89,90^ the coupling constants between electron and hole states would likewise be underestimated by the same amount, if not corrected as described below. On the other hand, as the energetic difference between hole states is much less affected by QP corrections, the Kohn–Sham and the quasi-particle coupling constants should be, in this case, much more aligned (similarly for vibrational coupling between electron states). In other words
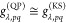
20should hold for the case where *p*, *q* are both electron or hole states. To test this conjecture and to get a more detailed picture, we consider the anthracene molecule again and compute the off-diagonal coupling constants  in the Kohn–Sham approximation analogous to eq 14, using the numerical settings from Section 3.2. To obtain the quasi-particle coupling constants  at G0W0 level of theory, we replace the Kohn–Sham eigenvalues in eq 14 by the corresponding quasi-particle energies. The results are summarized in Table 2, showing that the quasi-particle coupling constants between the hole states (and between the electron states) are relatively accurately described by the Kohn–Sham approximation with deviations of a few percent.

**Table 2:** Comparison of the G0W0 Off-Diagonal Coupling Constants and Those Obtained within the Kohn–Sham Approximation for the Electron and Hole States Closest to the Fermi Level and for Various Combinations *p*, *q*^a^

type of states	ν	*p*	*q*			
hole-	56	0	–1	0.4686	0.5140	–8.8
	55	–1	–2	0.3855	0.3805	–1.3
	52	0	–2	0.3912	0.4143	5.6
hole	48	0	–3	0.3374	0.3654	7.8
	54	–1	–3	0.3252	0.3491	–6.8
	45	–2	–3	0.2731	0.3061	–10.8
hole-	16	1	0	0.7325	0.5467	34.0
	22	1	–1	0.7613	0.6066	25.5
	56	1	–2	0.4873	0.3949	23.4
electron	36	2	0	1.5974	1.2702	25.8
	48	2	–2	1.2460	1.0500	18.7
	48	3	–1	1.1610	0.9711	19.5
electron-	56	1	2	0.5643	0.6053	–6.8
	52	1	3	0.3759	0.3796	–1.0
	51	1	4	0.3072	0.3072	0.0
electron	55	2	3	0.4648	0.4139	12.3
	54	2	4	0.2847	0.2647	7.6
	47	3	4	0.3935	0.3822	2.9

a Selection of modes ν is made for the strongest coupling constants between the considered state pairs (*p*, *q*). Last column shows the percentage deviation between the methods.

In contrast, when considering the coupling between electron and hole states, we notice significant deviations between  and  in Table 2 (≥18%), and propose the following correction scheme. The off-diagonal coupling matrix elements can easily be corrected, at least within the approximation eq 11 studied in this article, by just replacing the difference of the Kohn–Sham eigenvalues with the respective quasi-particle energy difference. In contrast to calculating all couplings in an expensive quasi-particle framework, this correction, based on a single G0W0 calculation at the equilibrium geometry, can be applied to all modes, which is a great simplification. The resulting corrections are summarized in Table 2.

In any event, even when this single G0W0 calculation is not feasible for more complex systems, our results suggest that at least within the electron sector (and hole sector), the off-diagonal coupling constants within the Kohn–Sham approximation are good approximations of their quasi-particle counterparts. These couplings are usually important for charge transport simulations.

### 3.3 Extended Numerical Comparison of Coupling Constants

The idea to use KS coupling constants for G0W0 coupling constants relies on the cancellation of terms and should be tested for more systems and more vibration modes. For this, we focus on small and medium-sized molecules. This focus also allows extending this benchmark to many-body approaches to the electron–phonon coupling constants, such as Δ*S*CF,^91^ multireference configuration interaction (MRCI)^92^ and second-order extended multiconfiguration quasi-degenerate perturbation theory (XMCQDPT2).^93^ We also examine approximate quasi-particles, i.e., the coupling constants obtained by approximations to the exact self-energy employing the G0W0^46^ and the Outer Valence Green’s function (OVGF)^44^ frameworks.

#### 3.3.1 Comparison of KS to G0W0 and Δ*S*CF Approaches

We first focus on pentacene, which has frequently been the subject of studies on electron–phonon interaction in the past.^18,72,94−96^ Specifically, we examine the lowest-energy hole (cationic) and electron (anionic) excitation of the neutral molecule and consider the relevant totally symmetric modes. For the comparison, we first compute the ground state equilibrium geometry of the neutral molecule by using the B3LYP hybrid functional^79−81^ and, subsequently, its normal modes. We employ TZV2P basis^75^ sets and GTH pseudopotentials.^85,86^ A finite difference approach to the normal modes is applied. As a starting point for the G0W0 procedure, we use the B3LYP orbitals and energies.

**Figure 3:**
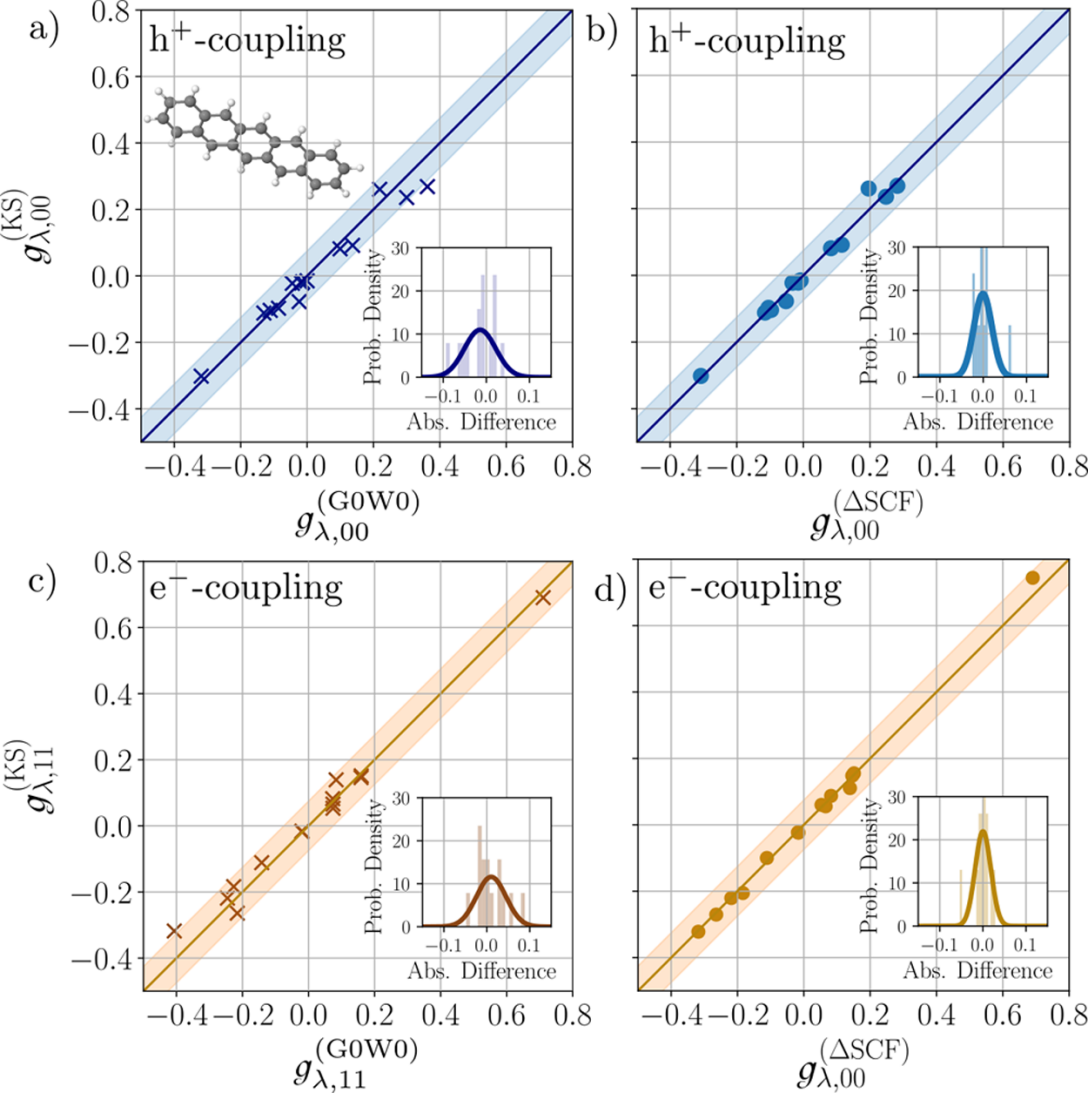
Comparison of the hole-phonon coupling constants in (a) and (b) and electron–phonon coupling constants in (c) and (d) for the molecule pentacene, computed with the KS, G0W0 and Δ*S*CF approach. Shaded areas reflect the interval ±0.075. Insets: Histogram of absolute differences and fitted normal distributions.

We compare the obtained KS and quasi-particle coupling constants from the G0W0 method for holes and electrons for each of the modes in Figure 3a,c, respectively, where each data point corresponds to one mode λ. We generally find that the KS and the G0W0 coupling constants align well, with only minor deviations for all the considered modes. To be more quantitative, we again consider the MAD value as a measure of agreement. For the MAD of the KS hole coupling constant (with respect to the G0W0 method), we obtain a value of 0.026 and a similar value of 0.025 for electrons. From these results, we conclude that the G0W0 method and the KS approach yield a good agreement between all the computed coupling constants, which is also reflected in the histograms of the absolute differences and the fitted normal distributions plotted in the insets of Figure 3a,c.

Alternatively to the G0W0 method, we compute the quasi-particle coupling constants from the total DFT energy difference, widely known as Δ*S*CF calculation.^91^ In the latter method, these parameters can be obtained from the change in the total energy of the ground state of the charged molecules with displaced geometries as . In the case of the lowest-energy hole state, the coupling constants can be determined with the help of eq 7 as
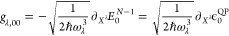
21and an analogous relation applies for electrons (). This Δ*S*CF approach to the coupling constants is to be distinguished from the KS approach based on the KS eigenvalues of DFT and provides a further, independent test. We take the same XC functional and the other numerical parameters for the G0W0 comparison.

The results for the lowest-energy hole- and electron–phonon couplings are plotted in Figure 3b,d, showing very good agreement. This qualitative result is also reflected in the MAD, which we determine for holes and electrons to be 0.013 and 0.011, respectively. Also, in this case, we conclude that the KS and many-body coupling (computed via Δ*S*CF) agree very well. In any event, this demonstrates that for pentacene, all the DFT-based methods that we considered here (KS, Δ*S*CF, and G0W0), although representing conceptually different approaches, give consistent electron–phonon coupling constants.

Note that, while this comparison establishes empirically the connection between KS and quasi-particle methods (G0W0) as well as to the many-body approaches (Δ*S*CF) for the lowest charged excitations, it remains to be investigated whether the agreement of the coupling constants arises from the common the basis of these approaches, i.e., the formulation based on a DFT reference, for the Δ*S*CF and the G0W0 method.

#### 3.3.2 Comparison to the Outer Valence Green’s Function Method

To address this question, we include the HF-based quasi-particle method OVGF,^44^ which is not based on the Kohn–Sham framework and allows us to examine whether the agreement observed in the previous subsection is attributable to the shared DFT basis of the methods employed. To this end, we consider the naphthalene molecule, for which previous work^97^ has provided reference coupling constants from OVGF. We optimize geometry and compute the normal modes using the CAM-B3LYP functional^82^ employing GTH pseudopotentials^85,86^ and cc-TZV2P basis sets.^75^ We identify the totally symmetric modes {*v*_1_, ···, *v*_7_}. The comparison of the mode energies of this assignment and those of ref (97) can be found in the Supporting Information Section S3.5.2. With some deviations for the mode *v*_5_, we generally find very good agreement between the normal mode energies of the modes from the two approaches.

**Figure 4:**
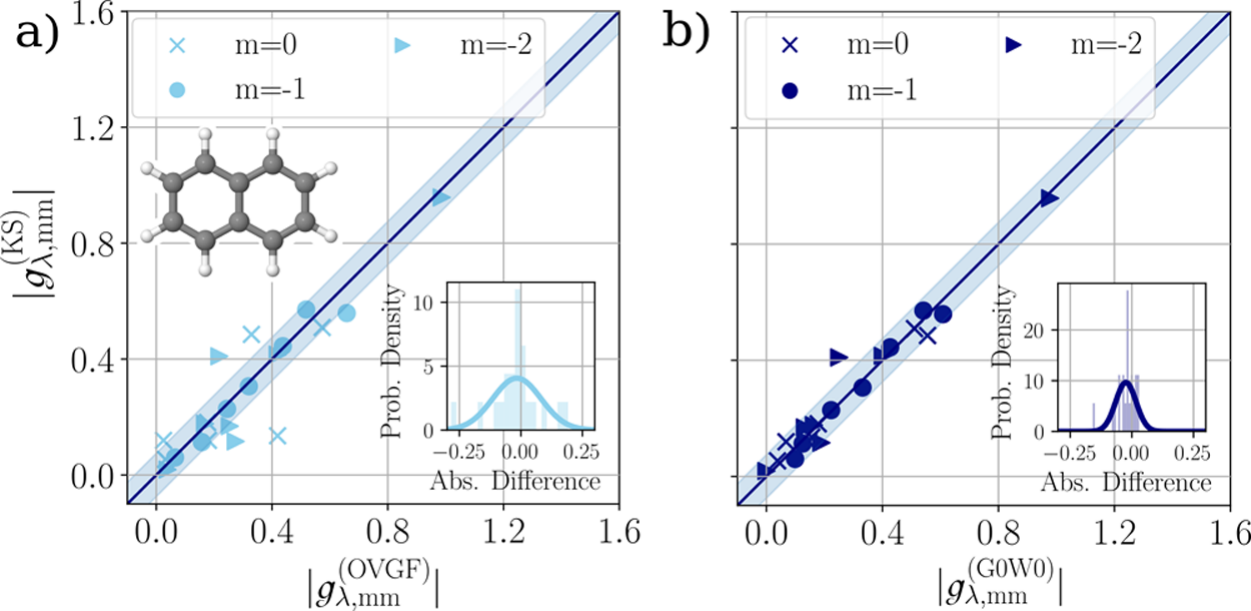
Comparison of diagonal hole-phonon-coupling constants of the molecule naphthalene. Compared are the absolute values for the first hole states (*m* ∈ {0, – 1, – 2}) between the results of HF-based QP method OVGF from ref (97) [(a), abscissa] to those of the CAM-B3LYP-cc [ordinate] and between G0W0 [(b), abscissa] and CAM-B3LYP-cc. Shaded area reflects the interval ±0.075. Insets: Histogram of absolute differences and fitted normal distributions.

Figure 4a compares the obtained CAM-B3LYP state-diagonal coupling constants of the first three hole states (*m* ∈ {0, – 1, – 2}) for the totally symmetric modes to those of ref (97) with OVGF. We restrict ourselves to these states, as the deeper lying hole states are less interesting and mutually closer in energy, so the unique identification of the states between the respective methods can not be guaranteed any more. The data is compiled in the Supporting Information Section S3.5.2.

The CAM-B3LYP coupling constants deviate noticeably from the OVGF results of ref (97), with MAD values for the states *m* = 0, – 1, – 2 of 0.0978, 0.0341, and 0.0710, respectively. To analyze the possible origin of the larger deviations, we verify that we get closer aligned results within the KS-based methods. For this reason, we also compute the coupling constants using the G0W0 method on top of the CAM-B3LYP treatment using the CAM-B3LYP normal modes and display the result in Figure 4b. As visible there, we find that the G0W0 coupling constants are much more aligned with the CAM-B3LYP values, which is reflected in the respective MAD values, which are calculated for the states *m* = 0, – 1, – 2 as 0.0267, 0.0254, and 0.0478 respectively. These values are pretty similar to the case of pentacene. This result, on the first glimpse, hints at some influence on the alignment of coupling constants when changing the formulation from a DFT to an HF reference method. However, a more detailed analysis of the deviations shows that especially the modes *v*_5_ and *v*_6_ stand out here, as they are the main cause of the MAD increase due to a redistribution of coupling strength to the state *m* = 0 (cf. Supporting Information Section S3.5.2 Table S10). This may be interpreted as a sign of slightly different normal mode directions *v*_5_ and *v*_6_ for the comparison. Unfortunately, we could not compare the mode pattern, as these are not published in ref (97). However, the deviation in mode frequency, in particular for mode *v*_5_ (cf. Supporting Information Section S3.5.2 Table S8), indicates a connection of these deviations to the mode(-pattern).

Even when excluding these modes, the OVGF diagonal coupling constants deviate slightly more strongly from their KS coupling constants than their G0W0 counterparts, as apparent from the respectively adjusted MAD values for *m* = 0, – 1, – 2 as 0.0490, 0.0380, and 0.0439. This suggests that the methodological basis has a small influence on these values. Nonetheless, these values do not deviate sufficiently from their G0W0 counterparts to make more definite conclusions, especially as other effects, e.g., basis sets, can have some influence at this scale. Accounting for these uncertainties, the computed coupling constants seem rather consistent.

**Figure 5:**
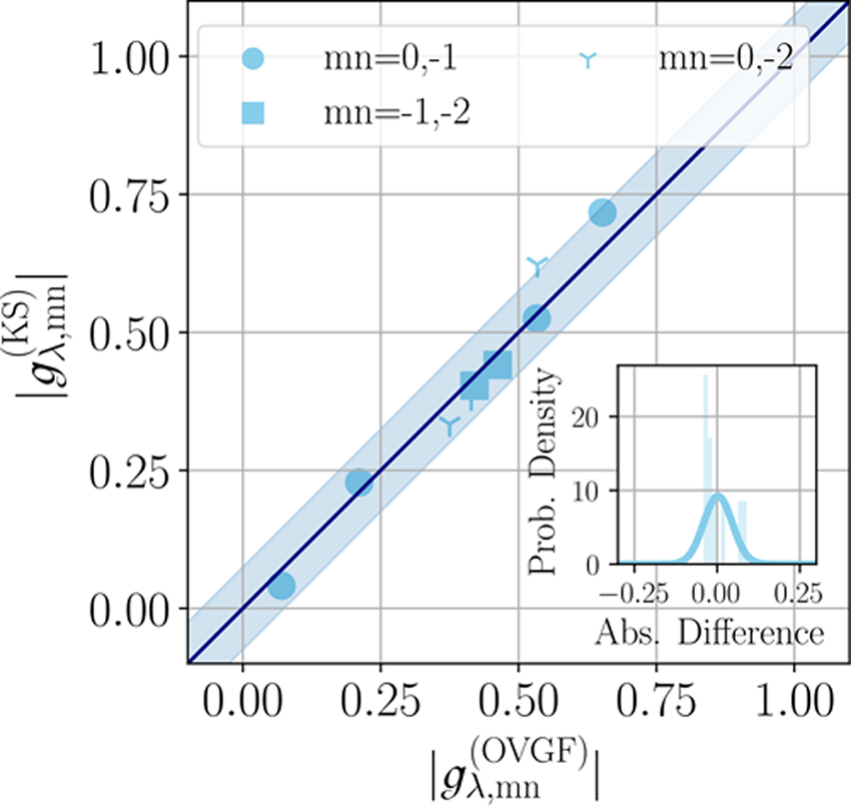
Comparison of off-diagonal hole-phonon-coupling constants for the first three charged excitations (*m* ∈ {0, – 1, – 2}) of the naphthalene molecule. Abscissa values were obtained via diabatization using the OVGF method [for details, see ref (97)]. Ordinate values are obtained from the derivative of KS eigenstates. We find very good agreement between the two methods. Shaded area reflects the interval ±0.075. Inset: Histogram of absolute differences and fitted normal distributions.

We extend this study to the off-diagonal vibrational coupling constants. Different ways exist to compute these for a given normal mode coordinate ref (97) computed this coupling by a local diabatization procedure at the conical intersection of the adiabatic potential energy surfaces,^98,99^ and thus from the adiabatic energies alone. It should be mentioned that the diabatization procedure does not generally allow the computation of the off-diagonal coupling constant for any given mode. For this reason, the comparison is restricted to those mode-state combinations, where ref (97) provides a reference value. We refrain from giving MAD values for this comparison, as only a selection of the possible coupling constants can be compared. Similar to our previous approach, we identify the respective modes by closeness in energy to those given in ref (97). The identified combinations are collected in the Supporting Information Section S3.5.2. The comparison between our values and theirs is depicted in Figure 5. We find that the off-diagonal coupling constants within the OVGF and the KS approaches are in much better agreement than the diagonal ones. The level of agreement is similar to the one between the G0W0 and KS approaches to the diagonal coupling constants.

While the relatively good agreement for the lowest-energy hole states of naphthalene between the methods, as demonstrated here, is promising, our results in this section also demonstrate possible limitations in the comparison of different approaches. Care is necessary if the underlying electronic structure or the phonon modes are not aligned enough between different methods. This is especially true in the case of (near-) degeneracies.

### 3.4 Exciton-Vibration Coupling Constants

#### 3.4.1 Exciton Coupling Constants from QP Coupling Constants

Having established that the KS scheme is, in principle, capable of successfully reproducing the QP coupling constants of charged, low-energy excitations, we now focus on the exciton–phonon coupling constants. Analogous to the multitude of different quasi-particle methods for the parametrization of the quasi-particle coupling constants, various (quasi-particle) approximations to the charge-neutral excitations exist. Consequently, this also translates to the vibrational coupling to these effective states. For example, one may employ BSE@GW^45,53,61−63^ approaches or linear-response time-dependent density functional theory (TDDFT),^100,101^ which both use a static approximation to the effective electron–hole interaction.^63^

We have to consider the effective interaction Ξ̂ introduced in eq 4 to simulate the exciton–phonon coupling. This interaction mixes independent electron–hole pair states and leads to the formation of excitons. In the quasi-particle description eq 12 of electron–hole pairs, the singlet exciton states can be exactly written as
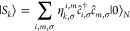
22where η are the exciton amplitudes. These states diagonalize the purely electronic part of the Hamiltonian eq 12,
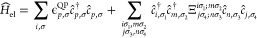
23As already briefly discussed in Section 2.4, in general, the interaction  may depend on the nuclear coordinates **R**, leading to an additional exciton–phonon coupling contribution, which cannot be inferred from the coupling of the holes and electrons alone. However, if this dependence is weak, which should be the case for sufficiently gapped systems, it can be neglected, as analyzed in Supporting Information Section S2. In this case, we can represent the two-particle, spin-singlet subspace of the effective Hamiltonian eq 12 in terms of the singlet eigenstates |*S*_*k*_⟩. It takes the form
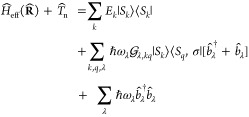
24Here, the exciton–phonon coupling constants  are solely determined from the QP coupling constants eq 13 and the exciton amplitudes η_*k*, σ_^*i*, *m*^ by a simple linear transformation. For convenience, we call this KSη method in the following. We emphasize that within the KSη approach, on top of the calculation of the vibrational couplings for the charged electron and hole states, a single calculation to obtain the exciton amplitudes at the equilibrium geometry is necessary to also obtain the vibrational coupling constants of the excitonic excitations.

#### 3.4.2 Comparison of KSη Method to Direct TD-DFT and XMCQDPT2 Approaches

In order to study this conjecture numerically, we focus again on pyrazine since the extensive literature on this molecule^35,84^ also includes studies of its singlet excitations by means of different many-body methods, which allows us to compare the coupling constants when excitonic excitations are considered. We use (linear-response) TD-DFT,^100,102^ as implemented in CP2K,^75^ to access the lowest 15 singlet excited states using the CAM-B3LYP functional^82^ and employing the cc-TZV2P basis set and GTH-pseudopotentials^85,86^ at the charge-neutral equilibrium geometry. The CAM-B3LYP coupling constants for electrons and holes from above are further used to set up the effective Hamiltonian. Both are combined to obtain the excited states coupling constants  as described above.

**Figure 6:**
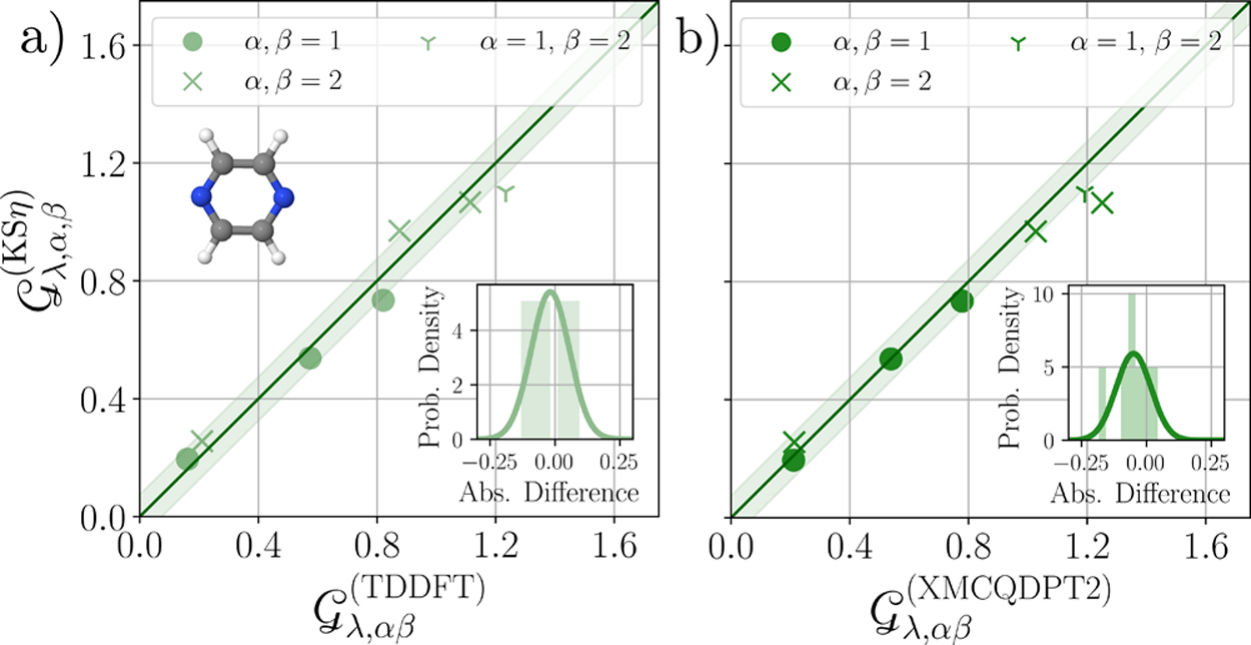
Comparison of the diagonal and off-diagonal exciton–phonon coupling constants for the molecule pyrazine for the first two excited singlet states (*S*_1_, *S*_2_). KSη coupling constants are compared to the values of the direct approach by application of TD-DFT in (a) and to the results of ref (84) obtained by XMCQDPT2 in (b). Shaded area reflects the interval ±0.075. Insets: Histogram of absolute differences and fitted normal distributions.

Alternatively, to the KSη approach, we access the vibrational coupling constants of the excited states directly from the TD-DFT calculations as a discretized derivative of the energies of the excited states along the relevant normal modes ν_6*a*_, ν_1_, ν_9*a*_ and ν_10*a*_. This requires a local diabatization procedure, analogously to the one discussed in Supporting Information Section S3.4.2. From the analysis of the states and comparison with the literature, it becomes clear that TD-DFT does, in this case, not correctly reproduce the state ordering.^103,104^ Hence, we identify the respective states by examining the symmetry of the transitions. Based on this, we obtain TD-DFT coupling constants,
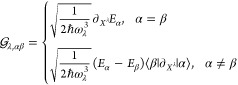
25which are plotted against the KSη coupling constants in Figure 6a. We find good agreement within a small expected range of numerical errors, which reveals that the exciton–phonon coupling constants can be efficiently obtained from the QP coupling constants (described at the KS level) for pyrazine. This result confirms that for sufficiently gapped systems, the **R**-dependence of the effective electron–hole interaction is negligible.

To have an independent benchmark for these coupling constants, we compare our results to the results of ref (84), which have employed the extended multiconfiguration quasi-degenerate second-order perturbation theory^93^ (XMCQDPT2). One might anticipate that the accuracy of the resulting exciton–phonon coupling constants may be reduced by effects that also lead to incorrect state ordering. However, as visible in Figure 6b, we obtain very good agreement with this “higher level” quantum chemistry method. We attribute this to the insignificance of a constant energy offset when calculating the coupling constants. This further corroborates that the Kohn–Sham DFT-based determination via quasi-particle coupling constants and exciton amplitudes η can, in principle, reproduce exciton–phonon coupling constants from other quantum chemistry methods.

## 4 Discussion

For large molecules or periodic structures, a DFT approach is often the only numerically accessible way to address electron–phonon coupling but has no intrinsic justification. From this perspective, the results for the coupling constants of the low-energy excitations (charged and neutral) presented above are promising. On the other hand, applying this KS approach requires good knowledge of limitations and the development of indicators for its reliability. Other important factors, including state order and energetic neighborhood relations, codetermine the physics of the effective model eq 12. Our contribution here addressed the relationship between the different methods from the analytic and numerical perspectives, which was made possible by studying smaller, accessible systems. The presented examples showed that the Kohn–Sham approximation could, despite strong variations in KS eigenvalues, reproduce low-energy quasi-particle and many-body coupling constants for charged excitations and, with the KSη approach, also for charge-neutral excitations.

However, some limitations should be mentioned here, where an unreflected application can lead to erroneous results: First, the electronic states obtained within a certain DFT framework are not guaranteed to provide a sufficiently accurate approximation to the quasi-particle orbitals. For example, this can be questioned for the application of semilocal functionals to mixed-valence compounds, which should also result in inconsistent coupling constants.^105^ Second, even beyond this, the quasi-particle picture will break down for states requiring a many-body treatment. This may be the case for transition metal oxides.^63^ In this case, the quasi-particle method becomes inaccurate or fails; thus, the Kohn–Sham approximation is not expected to yield meaningful results.

Since it is a priori unclear which level of theory is appropriate for computing the electron–phonon coupling constants in a specific system, prior works have also compared different DFT flavors and quasi-particle methods.^106,107^ However, these studies are still rare and often limited to diagonal coupling constants. Also, despite some pioneering work for C60 molecules,^108^ there is a lack of comparisons for larger molecular systems or periodic structures. Under the impression that DFT is, to date, the most widespread method to calculate vibrational couplings, this lack of reference data emphasizes the need for more comparisons, especially when the goal is to give guidelines for its reliable application. We hope that our work sparks further activity in this direction.

## Supporting Information Available

Derivation of the effective Hamiltonian, analytical considerations on nuclear dependence of the effective interaction, technical details, benchmarks, and data underlying the figures of the main manuscript (PDF)

## References

[ref1] Bardeen J.; Cooper L. N.; Schrieffer J. R. Microscopic Theory of Superconductivity. Phys. Rev. 1957, 106, 162–164. 10.1103/PhysRev.106.162.

[ref2] Bardeen J.; Cooper L. N.; Schrieffer J. R. Theory of Superconductivity. Phys. Rev. 1957, 108, 1175–1204. 10.1103/PhysRev.108.1175.

[ref3] Nambu Y. Quasi-Particles and Gauge Invariance in the Theory of Superconductivity. Phys. Rev. 1960, 117, 648–663. 10.1103/PhysRev.117.648.

[ref4] Eliashberg G. M. Interactions between electrons and lattice vibrations in a superconductor. Sov. Phys. - JETP (Engl. Transl.) 1960, 11, 696-702.

[ref5] Budai J. D.; Hong J.; Manley M. E.; Specht E. D.; Li C. W.; Tischler J. Z.; Abernathy D. L.; Said A. H.; Leu B. M.; Boatner L. A.; McQueeney R. J.; Delaire O. Metallization of vanadium dioxide driven by large phonon entropy. Nature 2014, 515, 535–539. 10.1038/nature13865. 25383524

[ref6] Bansal D.; Niedziela J. L.; Calder S.; Lanigan-Atkins T.; Rawl R.; Said A. H.; Abernathy D. L.; Kolesnikov A. I.; Zhou H.; Delaire O. Magnetically driven phonon instability enables the metal–insulator transition in h-FeS. Nat. Phys. 2020, 16, 669–675. 10.1038/s41567-020-0857-1.

[ref7] Luo H.; Gao Q.; Liu H.; Gu Y.; Wu D.; Yi C.; Jia J.; Wu S.; Luo X.; Xu Y.; Zhao L.; Wang Q.; Mao H.; Liu G.; Zhu Z.; Shi Y.; Jiang K.; Hu J.; Xu Z.; Zhou X. J. Electronic nature of charge density wave and electron-phonon coupling in kagome superconductor KV3Sb5. Nat. Commun. 2022, 13, 273 10.1038/s41467-021-27946-6. 35022418 PMC8755796

[ref8] Ziman J. Electrons and Phonons: The Theory of Transport Phenomena in Solids; Oxford University Press, 2001.

[ref9] Liao B.; Qiu B.; Zhou J.; Huberman S.; Esfarjani K.; Chen G. Significant Reduction of Lattice Thermal Conductivity by the Electron-Phonon Interaction in Silicon with High Carrier Concentrations: A First-Principles Study. Phys. Rev. Lett. 2015, 114, 115901 10.1103/PhysRevLett.114.115901. 25839292

[ref10] Zhou J.; Shin H. D.; Chen K.; Song B.; Duncan R. A.; Xu Q.; Maznev A. A.; Nelson K. A.; Chen G. Direct observation of large electron–phonon interaction effect on phonon heat transport. Nat. Commun. 2020, 11, 6040 10.1038/s41467-020-19938-9. 33247148 PMC7695728

[ref11] Brédas J.; Beljonne D.; Coropceanu V.; Cornil J. Charge-transfer and energy-transfer processes in pi-conjugated oligomers and polymers:: A molecular picture. Chem. Rev. 2004, 104, 4971–5003. 10.1021/cr040084k. 15535639

[ref12] Hestand N. J.; Spano F. C. Expanded Theory of H- and J-Molecular Aggregates: The Effects of Vibronic Coupling and Intermolecular Charge Transfer. Chem. Rev. 2018, 118, 7069–7163. 10.1021/acs.chemrev.7b00581. 29664617

[ref13] Spies L.; Biewald A.; Fuchs L.; Merkel K.; Righetto M.; Xu Z.; Guntermann R.; Hooijer R.; Herz L. M.; Ortmann F.; Schneider J.; Bein T.; Hartschuh A. Spatiotemporal Spectroscopy of Fast Excited-State Diffusion in 2D Covalent Organic Framework Thin Films. J. Am. Chem. Soc. 2025, 147, 1758–1766. 10.1021/jacs.4c13129. 39746155

[ref14] Brédas J. L.; Calbert J. P.; da Silva Filho D. A.; Cornil J. Organic semiconductors: A theoretical characterization of the basic parameters governing charge transport. Proc. Natl. Acad. Sci. U. S. A. 2002, 99, 5804–5809. 10.1073/pnas.092143399. 11972059 PMC122857

[ref15] Coropceanu V.; Cornil J.; da Silva Filho D. A.; Olivier Y.; Silbey R.; Brédas J.-L. Charge Transport in Organic Semiconductors. Chem. Rev. 2007, 107, 926–952. 10.1021/cr050140x. 17378615

[ref16] Ortmann F.; Bechstedt F.; Hannewald K. Theory of charge transport in organic crystals: Beyond Holstein’s small-polaron model. Phys. Rev. B 2009, 79, 235206 10.1103/PhysRevB.79.235206.

[ref17] Fratini S.; Ciuchi S. Bandlike Motion and Mobility Saturation in Organic Molecular Semiconductors. Phys. Rev. Lett. 2009, 103, 266601 10.1103/PhysRevLett.103.266601. 20366327

[ref18] Hutsch S.; Panhans M.; Ortmann F. Charge carrier mobilities of organic semiconductors: ab initio simulations with mode-specific treatment of molecular vibrations. npj Comput. Mater. 2022, 8, 228 10.1038/s41524-022-00915-3.

[ref19] Hutsch S.; Ortmann F. Impact of heteroatoms and chemical functionalisation on crystal structure and carrier mobility of organic semiconductors. npj Comput. Mater. 2024, 10, 206 10.1038/s41524-024-01397-1.

[ref20] Zhang X. B.; Taliercio T.; Kolliakos S.; Lefebvre P. Influence of electron-phonon interaction on the optical properties of III nitride semiconductors. J. Phys.: Condens. Matter 2001, 13, 7053 10.1088/0953-8984/13/32/312.

[ref21] Heinemeyer U.; Scholz R.; Gisslén L.; Alonso M. I.; Ossó J. O.; Garriga M.; Hinderhofer A.; Kytka M.; Kowarik S.; Gerlach A.; Schreiber F. Exciton-phonon coupling in diindenoperylene thin films. Phys. Rev. B 2008, 78, 085210 10.1103/PhysRevB.78.085210.

[ref22] Benduhn J.; Tvingstedt K.; Piersimoni F.; Ullbrich S.; Fan Y.; Tropiano M.; McGarry K. A.; Zeika O.; Riede M. K.; Douglas C. J.; Barlow S.; Marder S. R.; Neher D.; Spoltore D.; Vandewal K. Intrinsic non-radiative voltage losses in fullerene-based organic solar cells. Nat. Energy 2017, 2, 17053 10.1038/nenergy.2017.53.

[ref23] Panhans M.; Hutsch S.; Benduhn J.; Schellhammer K. S.; Nikolis V. C.; Vangerven T.; Vandewal K.; Ortmann F. Molecular vibrations reduce the maximum achievable photovoltage in organic solar cells. Nat. Commun. 2020, 11, 1488 10.1038/s41467-020-15215-x. 32198376 PMC7083957

[ref24] Chen X.-K.; Qian D.; Wang Y.; Kirchartz T.; Tress W.; Yao H.; Yuan J.; Hülsbeck M.; Zhang M.; Zou Y.; Sun Y.; Li Y.; Hou J.; Inganäs O.; Coropceanu V.; Bredas J.-L.; Gao F. A unified description of non-radiative voltage losses in organic solar cells. Nature Energy 2021, 6, 799–806. 10.1038/s41560-021-00843-4.

[ref25] Born M.; Oppenheimer R. Zur Quantentheorie der Molekülen. Annalen der Physik 1927, 389, 457–484. 10.1002/andp.19273892002.

[ref26] Parrinello M.; Rahman A. Crystal Structure and Pair Potentials: A Molecular-Dynamics Study. Phys. Rev. Lett. 1980, 45, 1196–1199. 10.1103/PhysRevLett.45.1196.

[ref27] Parrinello M.; Rahman A. Polymorphic transitions in single crystals: A new molecular dynamics method. J. Appl. Phys. 1981, 52, 7182–7190. 10.1063/1.328693.

[ref28] Car R.; Parrinello M. Unified Approach for Molecular Dynamics and Density-Functional Theory. Phys. Rev. Lett. 1985, 55, 2471–2474. 10.1103/PhysRevLett.55.2471. 10032153

[ref29] Krack M.; Parrinello M. All-electron ab-initio molecular dynamics. Phys. Chem. Chem. Phys. 2000, 2, 2105–2112. 10.1039/b001167n.

[ref30] Kühne T. D.; Krack M.; Mohamed F. R.; Parrinello M. Efficient and Accurate Car-Parrinello-like Approach to Born-Oppenheimer Molecular Dynamics. Phys. Rev. Lett. 2007, 98, 066401 10.1103/PhysRevLett.98.066401. 17358962

[ref31] Marx D.; Hutter J. Ab Initio Molecular Dynamics: Basic Theory and Advanced Methods; Cambridge University Press, 2009.

[ref32] Worth G. A.; Cederbaum L. S. BEYOND BORN-OPPENHEIMER: Molecular Dynamics Through a Conical Intersection. Annu. Rev. Phys. Chem. 2004, 55, 127–158. 10.1146/annurev.physchem.55.091602.094335. 15117250

[ref33] Köppel H.; Domcke W.; Cederbaum L. S. Advances in Chemical Physics; John Wiley & Sons, Ltd, 1984; pp. 59–246.

[ref34] Köppel H. Conical Intersections; World Scientific, 2004; pp. 175–204.

[ref35] Woywod C.; Domcke W.; Sobolewski A. L.; Werner H. Characterization of the S1–S2 conical intersection in pyrazine using ab initio multiconfiguration self-consistent-field and multireference configuration-interaction methods. J. Chem. Phys. 1994, 100, 1400–1413. 10.1063/1.466618.

[ref36] Schuurman M. S.; Yarkony D. R. On the vibronic coupling approximation: A generally applicable approach for determining fully quadratic quasidiabatic coupled electronic state Hamiltonians. J. Chem. Phys. 2007, 127, 094104 10.1063/1.2756540. 17824729

[ref37] Lévêque C.; Komainda A.; Taïeb R.; Köppel H. Ab initio quantum study of the photodynamics and absorption spectrum for the coupled 11A2 and 11B1 states of SO2. J. Chem. Phys. 2013, 138, 044320 10.1063/1.4776758. 23387597

[ref38] Subotnik J. E.; Alguire E. C.; Ou Q.; Landry B. R.; Fatehi S. The Requisite Electronic Structure Theory To Describe Photoexcited Nonadiabatic Dynamics: Nonadiabatic Derivative Couplings and Diabatic Electronic Couplings. Acc. Chem. Res. 2015, 48, 1340–1350. 10.1021/acs.accounts.5b00026. 25932499

[ref39] Plasser F.; Gómez S.; Menger M. F. S. J.; Mai S.; González L. Highly efficient surface hopping dynamics using a linear vibronic coupling model. Phys. Chem. Chem. Phys. 2019, 21, 57–69. 10.1039/C8CP05662E. 30306987

[ref40] Aleotti F.; Aranda D.; Yaghoubi Jouybari M.; Garavelli M.; Nenov A.; Santoro F. Parameterization of a linear vibronic coupling model with multiconfigurational electronic structure methods to study the quantum dynamics of photoexcited pyrene. J. Chem. Phys. 2021, 154, 104106 10.1063/5.0044693. 33722019

[ref41] Hohenberg P.; Kohn W. Inhomogeneous Electron Gas. Phys. Rev. 1964, 136, B864–B871. 10.1103/PhysRev.136.B864.

[ref42] Kohn W.; Sham L. J. Self-Consistent Equations Including Exchange and Correlation Effects. Phys. Rev. 1965, 140, A1133–A1138. 10.1103/PhysRev.140.A1133.

[ref43] Giustino F. Electron-phonon interactions from first principles. Rev. Mod. Phys. 2017, 89, 015003 10.1103/RevModPhys.89.015003.

[ref44] von Niessen W.; Schirmer J.; Cederbaum L. S. Methods in Computational Molecular Physics; Diercksen G. H. F., Wilson S., Eds.; Springer Netherlands: Dordrecht, 1983; pp. 227–248.

[ref45] Hedin L. New Method for Calculating the One-Particle Green’s Function with Application to the Electron-Gas Problem. Phys. Rev. 1965, 139, A796–A823. 10.1103/PhysRev.139.A796.

[ref46] Wilhelm J.; Golze D.; Talirz L.; Hutter J.; Pignedoli C. A. Toward GW Calculations on Thousands of Atoms. J. Phys. Chem. Lett. 2018, 9, 306–312. 10.1021/acs.jpclett.7b02740. 29280376

[ref47] Cederbaum L. S.; Domcke W. A many-body approach to the vibrational structure in molecular electronic spectra. I. Theory. J. Chem. Phys. 1976, 64, 603–611. 10.1063/1.432250.

[ref48] Dyson F. J. The *S* Matrix in Quantum Electrodynamics. Phys. Rev. 1949, 75, 1736–1755. 10.1103/PhysRev.75.1736.

[ref49] Ortiz J. V. Brueckner orbitals, Dyson orbitals, and correlation potentials. Int. J. Quantum Chem. 2004, 100, 1131–1135. 10.1002/qua.20204.

[ref50] Ortiz J. V. Dyson-orbital concepts for description of electrons in molecules. J. Chem. Phys. 2020, 153, 070902 10.1063/5.0016472. 32828082

[ref51] Hüser F.; Olsen T.; Thygesen K. S. Quasiparticle GW calculations for solids, molecules, and two-dimensional materials. Phys. Rev. B 2013, 87, 235132 10.1103/PhysRevB.87.235132.

[ref52] Cederbaum L. S. Field Operators in Real Space. J. Phys. Chem. A 2016, 120, 3009–3014. 10.1021/acs.jpca.5b09444. 26594868

[ref53] Onida G.; Reining L.; Rubio A. Electronic excitations: density-functional versus many-body Green’s-function approaches. Rev. Mod. Phys. 2002, 74, 601–659. 10.1103/RevModPhys.74.601.

[ref54] Strinati G. Effects of dynamical screening on resonances at inner-shell thresholds in semiconductors. Phys. Rev. B 1984, 29, 5718–5726. 10.1103/PhysRevB.29.5718.

[ref55] Strinati G. Application of the Green’s functions method to the study of the optical properties of semiconductors. La Rivista del Nuovo Cimento (1978–1999) 1988, 11, 1–86. 10.1007/BF02725962.

[ref56] Liu J.; Asthana A.; Cheng L.; Mukherjee D. Unitary coupled-cluster based self-consistent polarization propagator theory: A third-order formulation and pilot applications. J. Chem. Phys. 2018, 148, 244110 10.1063/1.5030344. 29960360

[ref57] Schirmer J. Beyond the random-phase approximation: A new approximation scheme for the polarization propagator. Phys. Rev. A 1982, 26, 2395–2416. 10.1103/PhysRevA.26.2395.

[ref58] Schirmer J.; Cederbaum L. S.; Walter O. New approach to the one-particle Green’s function for finite Fermi systems. Phys. Rev. A 1983, 28, 1237–1259. 10.1103/PhysRevA.28.1237.

[ref59] Golze D.; Dvorak M.; Rinke P. The GW Compendium: A Practical Guide to Theoretical Photoemission Spectroscopy. Front. Chem. 2019, 7, 377 10.3389/fchem.2019.00377. 31355177 PMC6633269

[ref60] Luttinger J. M. Analytic Properties of Single-Particle Propagators for Many-Fermion Systems. Phys. Rev. 1961, 121, 942–949. 10.1103/PhysRev.121.942.

[ref61] Rohlfing M.; Louie S. G. Electron-hole excitations and optical spectra from first principles. Phys. Rev. B 2000, 62, 4927–4944. 10.1103/PhysRevB.62.4927.

[ref62] Bechstedt F. Many-Body Approach to Electronic Excitations; Springer-Verlag: Berlin Heidelberg, 2014.

[ref63] Martin R. M.; Reining L.; Ceperley D. M. Interacting Electrons - Theory and Computational Approaches, hardback ed.; Cambridge University Press: Cambridge, 2016.

[ref64] Ismail-Beigi S.; Louie S. G. Excited-State Forces within a First-Principles Green’s Function Formalism. Phys. Rev. Lett. 2003, 90, 076401 10.1103/PhysRevLett.90.076401. 12633253

[ref65] Faber C.; Boulanger P.; Attaccalite C.; Cannuccia E.; Duchemin I.; Deutsch T.; Blase X. Exploring approximations to the *GW* self-energy ionic gradients. Phys. Rev. B 2015, 91, 155109 10.1103/PhysRevB.91.155109.

[ref66] Foerster D.; Koval P.; Sánchez-Portal D. An O(N3) implementation of Hedin’s GW approximation for molecules. J. Chem. Phys. 2011, 135, 074105 10.1063/1.3624731. 21861554

[ref67] Govoni M.; Galli G. Large Scale GW Calculations. J. Chem. Theory Comput. 2015, 11, 2680–2696. 10.1021/ct500958p. 26575564

[ref68] Saito M. Electron-phonon coupling of electron- or hole-injected *C*_60_. Phys. Rev. B 2002, 65, 220508 10.1103/PhysRevB.65.220508.

[ref69] Iwahara N.; Sato T.; Tanaka K.; Chibotaru L. F. Vibronic coupling in *C*_60_^–^ anion revisited: Derivations from photoelectron spectra and DFT calculations. Phys. Rev. B 2010, 82, 245409 10.1103/PhysRevB.82.245409.

[ref70] Laflamme Janssen J.; Côté M.; Louie S. G.; Cohen M. L. Electron-phonon coupling in *C*_60_ using hybrid functionals. Phys. Rev. B 2010, 81, 073106 10.1103/PhysRevB.81.073106.

[ref71] Faber C.; Duchemin I.; Deutsch T.; Attaccalite C.; Olevano V.; Blase X. Electron–phonon coupling and charge-transfer excitations in organic systems from many-body perturbation theory. J. Mater. Sci. 2012, 47, 7472–7481. 10.1007/s10853-012-6401-7.

[ref72] Ortmann F.; Radke K. S.; Günther A.; Kasemann D.; Leo K.; Cuniberti G. Materials Meets Concepts in Molecule-Based Electronics. Adv. Funct. Mater. 2015, 25, 1933–1954. 10.1002/adfm.201402334.

[ref73] Hybertsen M. S.; Louie S. G. First-Principles Theory of Quasiparticles: Calculation of Band Gaps in Semiconductors and Insulators. Phys. Rev. Lett. 1985, 55, 1418–1421. 10.1103/PhysRevLett.55.1418. 10031814

[ref74] Ku W.; Eguiluz A. G. Band-Gap Problem in Semiconductors Revisited: Effects of Core States and Many-Body Self-Consistency. Phys. Rev. Lett. 2002, 89, 126401 10.1103/PhysRevLett.89.126401. 12225107

[ref75] Kühne T. D.; Iannuzzi M.; Del Ben M.; Rybkin V. V.; Seewald P.; Stein F.; Laino T.; Khaliullin R. Z.; Schütt O.; Schiffmann F.; Golze D.; Wilhelm J.; Chulkov S.; Bani-Hashemian M. H.; Weber V.; Borštnik U.; Taillefumier M.; Jakobovits A. S.; Lazzaro A.; Pabst H.; Müller T.; Schade R.; Guidon M.; Andermatt S.; Holmberg N.; Schenter G. K.; Hehn A.; Bussy A.; Belleflamme F.; Tabacchi G.; Glöß A.; Lass M.; Bethune I.; Mundy C. J.; Plessl C.; Watkins M.; VandeVondele J.; Krack M.; Hutter J. CP2K: An electronic structure and molecular dynamics software package - Quickstep: Efficient and accurate electronic structure calculations. J. Chem. Phys. 2020, 152, 194103 10.1063/5.0007045. 33687235

[ref76] Perdew J. P.; Burke K.; Ernzerhof M. Generalized Gradient Approximation Made Simple. Phys. Rev. Lett. 1996, 77, 3865–3868. 10.1103/PhysRevLett.77.3865. 10062328

[ref77] Perdew J. P.; Ernzerhof M.; Burke K. Rationale for mixing exact exchange with density functional approximations. J. Chem. Phys. 1996, 105, 9982–9985. 10.1063/1.472933.

[ref78] Adamo C.; Barone V. Toward reliable density functional methods without adjustable parameters: The PBE0 model. J. Chem. Phys. 1999, 110, 6158–6170. 10.1063/1.478522.

[ref79] Becke A. D. Density-functional exchange-energy approximation with correct asymptotic behavior. Phys. Rev. A 1988, 38, 3098–3100. 10.1103/PhysRevA.38.3098. 9900728

[ref80] Lee C.; Yang W.; Parr R. G. Development of the Colle-Salvetti correlation-energy formula into a functional of the electron density. Phys. Rev. B 1988, 37, 785–789. 10.1103/PhysRevB.37.785. 9944570

[ref81] Vosko S. H.; Wilk L.; Nusair M. Accurate spin-dependent electron liquid correlation energies for local spin density calculations: a critical analysis. Can. J. Phys. 1980, 58, 1200–1211. 10.1139/p80-159.

[ref82] Yanai T.; Tew D. P.; Handy N. C. A new hybrid exchange–correlation functional using the Coulomb-attenuating method (CAM-B3LYP). Chem. Phys. Lett. 2004, 393, 51–57. 10.1016/j.cplett.2004.06.011.

[ref83] Seidner L.; Domcke W.; von Niessen W. *x*^2^*A*_G_---A^2^*B*_1g_ conical intersection in the pyrazine cation and its effect on the photoelectron spectrum. Chem. Phys. Lett. 1993, 205, 117–122. 10.1016/0009-2614(93)85176-O.

[ref84] Sala M.; Saab M.; Lasorne B.; Gatti F.; Guérin S. Laser control of the radiationless decay in pyrazine using the dynamic Stark effect. J. Chem. Phys. 2014, 140, 194309 10.1063/1.4875736. 24852540

[ref85] Goedecker S.; Teter M.; Hutter J. Separable dual-space Gaussian pseudopotentials. Phys. Rev. B 1996, 54, 1703–1710. 10.1103/PhysRevB.54.1703. 9986014

[ref86] Hartwigsen C.; Goedecker S.; Hutter J. Relativistic separable dual-space Gaussian pseudopotentials from H to Rn. Phys. Rev. B 1998, 58, 3641–3662. 10.1103/PhysRevB.58.3641. 9986014

[ref87] Del Ben M.; Hutter J.; VandeVondele J. Electron Correlation in the Condensed Phase from a Resolution of Identity Approach Based on the Gaussian and Plane Waves Scheme. J. Chem. Theory Comput. 2013, 9, 2654–2671. 10.1021/ct4002202. 26583860

[ref88] Del Ben M.; Schütt O.; Wentz T.; Messmer P.; Hutter J.; VandeVondele J. Enabling simulation at the fifth rung of DFT: Large scale RPA calculations with excellent time to solution. Comput. Phys. Commun. 2015, 187, 120–129. 10.1016/j.cpc.2014.10.021.

[ref89] Sham L. J.; Schlüter M. Density-Functional Theory of the Energy Gap. Phys. Rev. Lett. 1983, 51, 1888–1891. 10.1103/PhysRevLett.51.1888.

[ref90] Perdew J. P.; Levy M. Physical Content of the Exact Kohn-Sham Orbital Energies: Band Gaps and Derivative Discontinuities. Phys. Rev. Lett. 1983, 51, 1884–1887. 10.1103/PhysRevLett.51.1884.

[ref91] Jones R. O.; Gunnarsson O. The density functional formalism, its applications and prospects. Rev. Mod. Phys. 1989, 61, 689–746. 10.1103/RevModPhys.61.689.

[ref92] Werner H.; Knowles P. J. An efficient internally contracted multiconfiguration–reference configuration interaction method. J. Chem. Phys. 1988, 89, 5803–5814. 10.1063/1.455556.

[ref93] Granovsky A. A. Extended multi-configuration quasi-degenerate perturbation theory: The new approach to multi-state multi-reference perturbation theory. J. Chem. Phys. 2011, 134, 214113 10.1063/1.3596699. 21663350

[ref94] Hatch R. C.; Huber D. L.; Höchst H. Electron-Phonon Coupling in Crystalline Pentacene Films. Phys. Rev. Lett. 2010, 104, 047601 10.1103/PhysRevLett.104.047601. 20366738

[ref95] Yi Y.; Coropceanu V.; Brédas J.-L. Nonlocal electron-phonon coupling in the pentacene crystal: Beyond the Γ-point approximation. J. Chem. Phys. 2012, 137, 164303 10.1063/1.4759040. 23126706

[ref96] Sato H.; Abd Rahman S. A.; Yamada Y.; Ishii H.; Yoshida H. Conduction band structure of high-mobility organic semiconductors and partially dressed polaron formation. Nat. Mater. 2022, 21, 910–916. 10.1038/s41563-022-01308-z. 35851148

[ref97] Ghanta S.; Reddy V. S.; Mahapatra S. Theoretical study of electronically excited radical cations of naphthalene and anthracene as archetypal models for astrophysical observations. Part I. Static aspects. Phys. Chem. Chem. Phys. 2011, 13, 14523–14530. 10.1039/c1cp21083a. 21750790

[ref98] Baer M. Adiabatic and diabatic representations for atom-molecule collisions: Treatment of the collinear arrangement. Chem. Phys. Lett. 1975, 35, 112–118. 10.1016/0009-2614(75)85599-0.

[ref99] Mead C. A.; Truhlar D. G. Conditions for the definition of a strictly diabatic electronic basis for molecular systems. J. Chem. Phys. 1982, 77, 6090–6098. 10.1063/1.443853.

[ref100] Casida M. E.; Jamorski C.; Casida K. C.; Salahub D. R. Molecular excitation energies to high-lying bound states from time-dependent density-functional response theory: Characterization and correction of the time-dependent local density approximation ionization threshold. J. Chem. Phys. 1998, 108, 4439–4449. 10.1063/1.475855.

[ref101] Vasiliev I.; Ögüt S.; Chelikowsky J. R. Ab Initio Excitation Spectra and Collective Electronic Response in Atoms and Clusters. Phys. Rev. Lett. 1999, 82, 1919–1922. 10.1103/PhysRevLett.82.1919.

[ref102] Casida M. E. Recent Advances in Density Functional Methods; World Scientific; 1995, pp. 155–192.

[ref103] Werner U.; Mitrić R.; Suzuki T.; Bonačić-Koutecký V. Nonadiabatic dynamics within the time dependent density functional theory: Ultrafast photodynamics in pyrazine. Chem. Phys. 2008, 349, 319–324. 10.1016/j.chemphys.2008.02.061.

[ref104] Jacquemin D.; Wathelet V.; Perpète E. A.; Adamo C. Extensive TD-DFT Benchmark: Singlet-Excited States of Organic Molecules. J. Chem. Theory Comput. 2009, 5, 2420–2435. 10.1021/ct900298e. 26616623

[ref105] Vandewal K.; Benduhn J.; Schellhammer K. S.; Vangerven T.; Rückert J. E.; Piersimoni F.; Scholz R.; Zeika O.; Fan Y.; Barlow S.; Neher D.; Marder S. R.; Manca J.; Spoltore D.; Cuniberti G.; Ortmann F. Absorption Tails of Donor:C60 Blends Provide Insight into Thermally Activated Charge-Transfer Processes and Polaron Relaxation. J. Am. Chem. Soc. 2017, 139, 1699–1704. 10.1021/jacs.6b12857. 28068763

[ref106] Wong Z. C.; Ungur L. Deriving the vibronic coupling constants of the cyclopentadienyl radical with density functional theory and GW. J. Chem. Phys. 2020, 153, 064303 10.1063/5.0014753. 35287446

[ref107] Wong Z. C.; Ungur L. Exploring vibronic coupling in the benzene radical cation and anion with different levels of the GW approximation. Phys. Chem. Chem. Phys. 2021, 23, 19054–19070. 10.1039/D1CP02795F. 34612443

[ref108] Faber C.; Janssen J. L.; Côté M.; Runge E.; Blase X. Electron-phonon coupling in the C_60_ fullerene within the many-body *GW* approach. Phys. Rev. B 2011, 84, 155104 10.1103/PhysRevB.84.155104.

