# Supplementary material for: Effective Electron-Vibration Coupling by Ab Initio Methods
Source: J Chem Theory Comput. 2025 Feb 24;21(5):2371–85. doi: 10.1021/acs.jctc.4c01608 (PMC11912215; doi:10.1021/acs.jctc.4c01608)
Supplement: Supplementary file 1 — ct4c01608_si_001.pdf [file ct4c01608_si_001.pdf]

Supporting Information: Effective Electron-Vibration Coupling by Ab Initio Methods

Maximilian F. X. Dorfner[†] and Frank Ortmann[†*]

[†] *TUM School of Natural Sciences, Technische Universität München, 85748 Garching b. München, Germany*

E-mail: frank.ortmann@tum.de

Contents

S1 Derivation of the effective Hamiltonian **3**

S1.1 The Hessian and the Normal Mode Coordinates 3

S1.2 Expansion of the Operators and Energies 6

S2 Nuclear Coordinate Dependence of the Effective Interaction **8**

S3 From Quasi-Particles to a Practical Scheme **11**

S3.1 Technical Details of the Computations 11

S3.2 Functional Dependence of the KS Coupling Constants 13

S3.2.1 Normal Modes 14

S3.2.2 Kohn-Sham Eigenvalues at the Equilibrium Geometry 14

S3.2.3 Diagonal Coupling Constants 15

S3.2.4 Off-Diagonal Coupling Constants 15

S3.3 Basis Set Dependence of the KS Coupling Constants 16
S3.4 Analytical and Numerical Analysis: QP vs. KS Coupling Constants 18
S3.4.1 Normal Modes 18
S3.4.2 Diabatization Procedure and Off-Diagonal Coupling 19
S3.5 Extended Numerical Comparison of Coupling Constants 21
S3.5.1 Comparison to G0W0 and ΔSCF 21
S3.5.2 Comparison to the Outer Valence Green's Function Method 21
S3.6 Exciton Coupling Constants 24
S3.6.1 Comparison to TD-DFT and XMCQDPT2 24

References **25**

S1 Derivation of the effective Hamiltonian

As motivated in the main manuscript, we study the effective Hamiltonian

$$\begin{aligned}\hat{H}_{\text{eff}}(\mathbf{R}) = E_0^N\Big(\mathbf{R}\Big) \quad &+ \quad \sum_{p,\sigma} \epsilon_{p,\sigma}^{\text{QP}}\Big(\mathbf{R}\Big)\hat{c}_{p,\sigma}^{\dagger}\Big(\mathbf{R}\Big)\hat{c}_{p,\sigma}\Big(\mathbf{R}\Big) \\ &+ \sum_{\substack{i\sigma_1,m\sigma_2 \\ j\sigma_3,n\sigma_4}} \hat{c}_{i,\sigma_1}^{\dagger}\hat{c}_{m,\sigma_2}^{\dagger}\Xi_{j\sigma_4;n\sigma_3}^{i\sigma_1;m\sigma_2}(\mathbf{R})\hat{c}_{n,\sigma_3}\hat{c}_{j,\sigma_4},\end{aligned} \tag{S1}$$

where $\hat{c}_{p,\sigma}^{\dagger}(\mathbf{R})$, $\hat{c}_{p,\sigma}(\mathbf{R})$ are fermionic creation and annihilation operators of the quasi-particle states $\{|p,\sigma\rangle\} = \{|i,\sigma\rangle\} \cup \{|m,\sigma\rangle\}$, where i,j labels the electron states and m,n the hole states that depend on the nuclear coordinate $\mathbf{R}$ with spin σ. Furthermore, $\epsilon_{p,\sigma}^{\text{QP}}(\mathbf{R})$ are the effective quasi-particle energies, and $\Xi_{j\sigma_4;n\sigma_3}^{i\sigma_1;m\sigma_2}(\mathbf{R})$ denote effective interaction matrix elements between the quasi-particles and quasi-holes. This effective Hamiltonian is constructed for the N particle ground state $|0\rangle_N$ with energy E_0^N. We introduce the Cartesian displacements $\mathbf{u}_s$ of atom s from a reference geometry $\mathbf{R}_0$ by

$$\mathbf{R}_s = \mathbf{R}_s^0 + \mathbf{u}_s, \tag{S2}$$

and consider that the dynamics will remain in a neighborhood of $\mathbf{R}^0$, which demands that the energy has a minimum at $\mathbf{R}^0$. To make use of this smallness of the displacements, we will expand all terms in Eq. (S1) that depend on the position $\mathbf{R}$ up to the first non-vanishing order in the Cartesian displacements

$$\mathbf{u} = \sum_{s,\alpha} u^{s,\alpha}\mathbf{e}_{s,\alpha}. \tag{S3}$$

S1.1 The Hessian and the Normal Mode Coordinates

We expand the first term in Eq. (S1), in the Cartesian displacements $\mathbf{u}$. With a vanishing linear term due to the minimum energy condition, we obtain up to the first non-vanishing

order in the Cartesian displacement

$$E_0\left(\mathbf{R}\right) \cong E_0\left(\mathbf{R}^0\right) + \frac{1}{2}\sum_{\substack{s_1,s_2\\ \alpha_1,\alpha_2}} u^{s_1,\alpha_1} \left.\frac{\partial^2 E_0\left(\mathbf{R}\right)}{\partial R^{s_1,\alpha_1}\partial R^{s_2,\alpha_2}}\right|_{\mathbf{R}^0} u^{s_2,\alpha_2}, \tag{S4}$$

where $R^{s,\alpha}$ is the αth coordinate of the sth atom and $u^{s,\alpha}$ are the respective Cartesian displacements.

Next, we introduce the components of the Hessian matrix $\underline{\mathbf{H}}$ by

$$H_{s_1,\alpha_1;s_2,\alpha_2} = \left.\frac{\partial^2 E_0\left(\mathbf{R}\right)}{\partial R^{s_1,\alpha_1}\partial R^{s_2,\alpha_2}}\right|_{\mathbf{R}^0}, \tag{S5}$$

and define the mass matrix $\underline{\mathbf{M}}$ by their matrix elements,

$$M_{s_1,\alpha_1;s_2,\alpha_2} = \delta_{s_1,s_2}\delta_{\alpha_1,\alpha_2}M_{s_1} \tag{S6}$$

which leads to the expansion of $\hat{H}_{\text{eff}} + \hat{T}_n$, as

$$\hat{H}_{\text{eff}} + \hat{T}_n = \sum_s \frac{\hat{\mathbf{P}}_s^2}{2M_s} + \frac{1}{2}\sum_{\substack{s_1,s_2\\ \alpha_1,\alpha_2}} u^{s_1,\alpha_1} H_{s_1,\alpha_1;s_2,\alpha_2} u^{s_2,\alpha_2} = \frac{1}{2}\hat{\mathbf{P}}^{\text{T}}\underline{\mathbf{M}}\hat{\mathbf{P}} + \frac{1}{2}\mathbf{u}^{\text{T}}\underline{\mathbf{H}}\mathbf{u}, \tag{S7}$$

where $\mathbf{P} = \sum_{s,\alpha} P_{s,\alpha}\mathbf{e}^{s,\alpha}$ is a coordinate vector of the canonical momentum. By introducing rescaled momenta

$$\tilde{\mathbf{P}} = \sqrt{\underline{\mathbf{M}}}^{-1}\mathbf{P} \tag{S8}$$

and rescaled displacements

$$\tilde{\mathbf{u}} = \sqrt{\underline{\mathbf{M}}}\mathbf{u} \tag{S9}$$

we can rewrite this result as

$$\hat{H}_{\text{eff}} + \hat{T}_n = \frac{1}{2}\tilde{\mathbf{P}}^{\text{T}}\tilde{\mathbf{P}} + \frac{1}{2}\tilde{\mathbf{u}}^{\text{T}} \underbrace{\sqrt{\underline{\mathbf{M}}}^{-1}\underline{\mathbf{H}}\sqrt{\underline{\mathbf{M}}}^{-1}}_{\equiv\underline{\tilde{\mathbf{H}}}} \tilde{\mathbf{u}}. \tag{S10}$$

The mass-weighted Hessian $\tilde{\mathbf{H}}$ is symmetric and positive semi-definite, i.e., there exists an orthogonal transformation $\underline{\mathbf{O}}$, such that $\underline{\tilde{\mathbf{H}}} = \underline{\mathbf{O}}^{\mathrm{T}}\underline{\Omega}\ \underline{\mathbf{O}}$, where $\underline{\Omega}$ is a diagonal matrix with only non-negative eigenvalues. By introducing new coordinates

$$\mathbf{X} = \underline{\mathbf{O}}\tilde{\mathbf{u}} \qquad \text{and} \qquad \mathbf{\Pi} = \underline{\mathbf{O}}^{\mathrm{T}}\tilde{\mathbf{P}}, \tag{S11}$$

we can further simplify the problem. The relation between the normal mode coordinates X^{λ}, with $\lambda \in \{1, ..., 3S\}$ and the Cartesian displacements are given by

$$X^{\lambda} = \sum_{s,\alpha} O^{\lambda}_{s,\alpha}\sqrt{M_s}u^{s,\alpha}, \tag{S12}$$

where M_s is the mass of the atoms s. Similarly, the relation between the respective conjugate momenta reads

$$\Pi_{\mu} = \sum_{s,\alpha} \frac{P_{s,\alpha}}{\sqrt{M_s}} O^{s,\alpha}_{\mu}, \tag{S13}$$

such that the canonical commutation relations,

$$\left[X^{\lambda}, \Pi_{\mu}\right] = i\hbar\delta^{\lambda}_{\mu},$$

are preserved. This transformation simplifies the Hamiltonian, as it decouples the nuclei part

$$\hat{H}_{\text{eff}} + \hat{T}_n = \frac{1}{2}\mathbf{\Pi}^{\mathrm{T}}\mathbf{\Pi} + \frac{1}{2}\mathbf{X}^{\mathrm{T}}\underline{\Omega}\ \mathbf{X} = \frac{1}{2}\sum_{\lambda}\left(\Pi_{\lambda}\Pi^{\lambda} + \omega_{\lambda}^{2}X_{\lambda}X^{\lambda}\right),$$

into $3S$ independent bosonic modes, where $\omega_{\lambda} = \sqrt{\underline{\Omega}_{\lambda\lambda}}$.

Introducing the creation and annihilation operators, by

$$\hat{b}_{\lambda} = \sqrt{\frac{\omega_{\lambda}}{2\hbar}}\left[X^{\lambda} + \frac{i}{\omega_{\lambda}}\Pi_{\lambda}\right], \quad \hat{b}^{\dagger}_{\lambda} = \sqrt{\frac{\omega_{\lambda}}{2\hbar}}\left[X^{\lambda} - \frac{i}{\omega_{\lambda}}\Pi_{\lambda}\right]$$

and the inverse relations

$$X^{\lambda} = \sqrt{\frac{\hbar}{2\omega_{\lambda}}} \left[\hat{b}_{\lambda}^{\dagger} + \hat{b}_{\lambda}\right], \quad \Pi_{\lambda} = i\sqrt{\frac{\hbar\omega_{\lambda}}{2}} \left[\hat{b}_{\lambda}^{\dagger} - \hat{b}_{\lambda}\right]$$

one finds the textbook result

$$\hat{H}_{\text{eff}} + \hat{T}_{n} = \sum_{\lambda} \hbar\omega_{\lambda} \left[\hat{b}_{\lambda}^{\dagger}\hat{b}_{\lambda} + 1/2\right]. \tag{S14}$$

S1.2 Expansion of the Operators and Energies

After the expansion of the ground state energy in terms of the normal modes, we next expand each of the remaining terms in Eq. (S1) up to the first non-vanishing order in the displacements around the equilibrium configuration. We apply the linearization operator, which can be written in different forms,

$$\sum_{s,\alpha} u^{s,\alpha} \frac{\partial}{\partial R^{s,\alpha}}(\cdots)\bigg|_{\mathbf{R}^0} = \sum_{\lambda} X^{\lambda} \frac{\partial}{\partial X^{\lambda}}(\cdots)\bigg|_{\mathbf{R}^0}, \tag{S15}$$

to expand the single-particle energies. In linear order, the expansion reads

$$\epsilon_{p,\sigma}^{\text{QP}}\left(\hat{\mathbf{R}}\right) = \epsilon_{p,\sigma}^{\text{QP}}\left(\mathbf{R}^0\right) + \sum_{s,\alpha} u^{s,\alpha} \frac{\partial \epsilon_{p,\sigma}^{\text{QP}}\left(\mathbf{R}\right)}{\partial R^{s,\alpha}}\bigg|_{\mathbf{R}^0} \tag{S16}$$

For the creation operators, one finds the relation

$$\hat{c}_{p,\sigma}^{\dagger}\left(\hat{\mathbf{R}}\right) = \hat{c}_{p,\sigma}^{\dagger}\left(\mathbf{R}^0\right) + \sum_{s,\alpha} u^{s,\alpha} \frac{\partial \hat{c}_{p,\sigma}^{\dagger}\left(\mathbf{R}\right)}{\partial R^{s,\alpha}}\bigg|_{\mathbf{R}^0} \tag{S17}$$

Next, we represent the creation operator for the single-particle state $|p,\sigma,\mathbf{R}\rangle$ at nuclei configuration $\mathbf{R}$, in terms of the single-particle states $|k,\sigma,\mathbf{R}^0\rangle$ of the configuration $\mathbf{R}^0$, which reads

$$\hat{c}_{p,\sigma}^{\dagger}\left(\mathbf{R}\right) = \sum_{q} \langle q,\sigma,\mathbf{R}^0|p,\sigma,\mathbf{R}\rangle \hat{c}_{q,\sigma}^{\dagger}\left(\mathbf{R}^0\right). \tag{S18}$$

Introducing the abbreviations

$$\epsilon^{\mathrm{QP}}_{p,\sigma} \equiv \epsilon^{\mathrm{QP}}_{p,\sigma}(\mathbf{R}^0), \quad E_0 \equiv E_0(\mathbf{R}^0), \quad \hat{c}^\dagger_{p,\sigma} \equiv \hat{c}^\dagger_{p,\sigma}(\mathbf{R}^0) \text{ and } \hat{c}_{p,\sigma} \equiv \hat{c}_{p,\sigma}(\mathbf{R}^0),$$

we write the Hamiltonian as

$$\begin{aligned}
\hat{H}_{\text{eff}} + \hat{T}_n \cong & \sum_\lambda \hbar\omega_\lambda \left[\hat{b}^\dagger_\lambda \hat{b}_\lambda + 1/2\right] \\
& + \sum_{p,\sigma} \left[\epsilon^{\mathrm{QP}}_{p,\sigma} + \sum_\lambda \sqrt{\frac{\hbar}{2\omega_\lambda}} \left[\hat{b}^\dagger_\lambda + \hat{b}_\lambda\right] \partial_{X^\lambda} \epsilon^{\mathrm{QP}}_{p,\sigma}\Big|_{\mathbf{R}^0}\right] \\
& \times \left[\hat{c}^\dagger_{p,\sigma} + \sum_{q,} \hat{c}^\dagger_{q,\sigma'} \sum_\lambda \sqrt{\frac{\hbar}{2\omega_\lambda}} \left[\hat{b}^\dagger_\lambda + \hat{b}_\lambda\right] \partial_{X^\lambda} \langle q,\sigma,\mathbf{R}^0|p,\sigma,\mathbf{R}\rangle\Big|_{\mathbf{R}^0}\right] \\
& \times \left[\hat{c}_{i,\sigma} + \sum_q \hat{c}_{q,\sigma} \sum_\lambda \sqrt{\frac{\hbar}{2\omega_\lambda}} \left[\hat{b}^\dagger_\lambda + \hat{b}_\lambda\right] \partial_{X^\lambda} \langle p,\sigma,\mathbf{R}|q,\sigma,\mathbf{R}^0\rangle\Big|_{\mathbf{R}^0}\right] \\
& + \sum_{\substack{i\sigma_1,m\sigma_2 \\ n\sigma_3,j\sigma_4}} \Xi^{i,\sigma_1;m,\sigma_2}_{n,\sigma_3;j\sigma_4} \hat{c}^\dagger_{i,\sigma_1} \hat{c}^\dagger_{m,\sigma_2} \hat{c}_{n,\sigma_3} \hat{c}_{j,\sigma_4},
\end{aligned} \tag{S19}$$

where we neglected the dependence of the effective interaction matrix elements on the nuclear coordinates $\mathbf{R}$ (see below). Using further the identity

$$\partial_{X^\lambda} \langle p,\sigma,\mathbf{R}|q,\sigma,\mathbf{R}^0\rangle\Big|_{\mathbf{R}^0} = -\partial_{X^\lambda} \langle p,\sigma,\mathbf{R}^0 \mid q,\sigma,\mathbf{R}\rangle\Big|_{\mathbf{R}^0}, \tag{S20}$$

and neglecting terms beyond leading order in the displacements, yields

$$\begin{aligned}
\hat{H}_{\text{eff}} + \hat{T}_n \cong & \sum_\lambda \hbar\omega_\lambda \left[\hat{b}^\dagger_\lambda \hat{b}_\lambda + 1/2\right] + \sum_{p,\sigma} \epsilon^{\mathrm{QP}}_{p,\sigma} \hat{c}^\dagger_{p,\sigma} \hat{c}_{p,\sigma} \\
& + \sum_{\substack{i\sigma_1,m\sigma_2 \\ j\sigma_3,n\sigma_4}} \hat{c}^\dagger_{i,\sigma_1} \hat{c}^\dagger_{m,\sigma_2} \Xi^{i\sigma_1;m\sigma_2}_{j\sigma_4;n\sigma_3} \hat{c}_{n,\sigma_3} \hat{c}_{j,\sigma_4} \\
& + \sum_{p,\sigma,\lambda} \sqrt{\frac{\hbar}{2\omega_\lambda}} \left[\hat{b}^\dagger_\lambda + \hat{b}_\lambda\right] \partial_{X^\lambda} \epsilon^{\mathrm{QP}}_{p,\sigma}\Big|_{\mathbf{R}^0} \hat{c}^\dagger_{p,\sigma} \hat{c}_{p,\sigma} \\
& + \sum_{p,q,\sigma,\lambda} (\epsilon^{\mathrm{QP}}_{q,\sigma} - \epsilon^{\mathrm{QP}}_{p,\sigma}) \partial_{X^\lambda} \langle p,\sigma,\mathbf{R}^0|q,\sigma,\mathbf{R}\rangle\Big|_{\mathbf{R}^0} \sqrt{\frac{\hbar}{2\omega_\lambda}} \left[\hat{b}^\dagger_\lambda + \hat{b}_\lambda\right] \hat{c}^\dagger_{p,\sigma} \hat{c}_{q,\sigma}.
\end{aligned}$$

After introducing the electron-phonon coupling constants

$$g_{\lambda,qp\sigma} = \begin{cases} \sqrt{\frac{1}{2\hbar\omega_\lambda^3}}\partial_{X^\lambda}\epsilon^{\mathrm{QP}}_{p,\sigma}, \quad p = q \\ \sqrt{\frac{1}{2\hbar\omega_\lambda^3}}\left(\epsilon^{\mathrm{QP}}_{q,\sigma} - \epsilon^{\mathrm{QP}}_{p,\sigma}\right)\langle p,\sigma,\mathbf{R}^0 \mid \partial_{X^\lambda} \mid q,\sigma,\mathbf{R}\rangle, p \neq q. \end{cases} \tag{S21}$$

one finds the final result

$$\begin{aligned} \hat{H}_{\mathrm{eff}} + \hat{T}_{\mathrm{n}} \;\; &\cong \;\; \sum_{p,\sigma}\epsilon^{\mathrm{QP}}_{p,\sigma}\hat{c}^\dagger_{p,\sigma}\hat{c}_{p,\sigma} + \sum_{p,\sigma,q,\lambda}\hbar\omega_\lambda g_{\lambda,pq\sigma}\left[\hat{b}^\dagger_\lambda + \hat{b}_\lambda\right]\hat{c}^\dagger_{p,\sigma}\hat{c}_{q,\sigma} \\ &+ \sum_{\substack{i\sigma_1,m\sigma_2 \\ j\sigma_3,n\sigma_4}}\hat{c}^\dagger_{i,\sigma_1}\hat{c}^\dagger_{m,\sigma_2}\Xi^{i\sigma_1;m\sigma_2}_{j\sigma_4;n\sigma_3}\hat{c}_{n,\sigma_3}\hat{c}_{j,\sigma_4} + \sum_\lambda \hbar\omega_\lambda\hat{b}^\dagger_\lambda\hat{b}_\lambda \end{aligned} \tag{S22}$$

in which the zero-point energy offset has been suppressed.

S2 Nuclear Coordinate Dependence of the Effective Interaction

As discussed in the main manuscript Sec. 2.4. and mentioned above, we neglect the **R**-dependence of the effective interaction Ξ in the case of sufficiently gapped systems. While this can be based on the empirical results referenced in Sec. 2.4. of the main manuscript, we also found an analytical scaling argument within the GW approximation that we want to present here.

Within the GW approximation, the effective electron-hole interaction matrix elements are given by,

$$\Xi^{p,\sigma_1;q,\sigma_2}_{k,\sigma_3;l\sigma_4} = \delta_{\sigma_1,\sigma_2}\delta_{\sigma_3,\sigma_4}2\overline{v}^{p;q}_{l;k}(\sigma_1,\sigma_2) - \delta_{\sigma_1,\sigma_4}\delta_{\sigma_2,\sigma_3}W^{p;q}_{k;l}(\sigma_1,\sigma_2),$$

where $\overline{v}^{p;q}_{l;k}(\sigma_1,\sigma_2)$ are matrix-elements of the unscreened (short-range) Coulomb operator $\hat{\overline{v}}$, i.e., where the $\mathbf{q} = 0$ component has been set to zero, and $W^{p;q}_{k;l}(\sigma_1,\sigma_2)$ are matrix elements

of the screened Coulomb operator $\hat{W}$.[1,2] As the unscreened short-range Coulomb operator cannot carry any nuclear coordinate dependence, the full dependence on nuclear coordinates enters the matrix elements only via the screened Coulomb operator. This screened Coulomb operator can be related[2] to the static limit of the time-ordered charge susceptibility $\hat{\chi} = \lim_{\omega \to 0} \hat{\chi}(\omega, \hat{\mathbf{R}})$, via

$$\hat{W} = \hat{v} + \hat{v} \circ \hat{\chi} \circ \hat{v}, \tag{S23}$$

where $\circ$ denotes a generalized matrix multiplication/convolution in orbital-spin space. In the space-time-domain, we can write down the susceptibility as

$$\hat{\chi}_{\sigma_1,\sigma_2}(\mathbf{r}_1, \mathbf{r}_2, t_1 - t_2) = -\frac{i}{\hbar} \langle \mathcal{T}_t \hat{n}_{\sigma_1}(\mathbf{r}_1, t_1) \hat{n}_{\sigma_2}(\mathbf{r}_2, t_2) \rangle + \frac{i}{\hbar} n_{\sigma_1}(\mathbf{r}_1) n_{\sigma_2}(\mathbf{r}_2). \tag{S24}$$

Here, $\hat{n}_\sigma(\mathbf{r}, t)$ denotes the density operator of spin-species σ at position $\mathbf{r}$ in the Heisenberg picture at time t, and $n_\sigma(\mathbf{r})$ is the spin density. Furthermore, $\mathcal{T}_t$ denotes the time-ordering operator. Using a canonical ensemble at $T = 0$ leads, in the case of a unique ground state, after Fourier transform to the frequency domain and, employing the Lehmann representation, to

$$\begin{aligned} \hat{\chi}_{\sigma_1,\sigma_2}(\mathbf{r}_1, \mathbf{r}_2, \omega) &= \sum_\alpha \frac{{}_N\langle 0 | \hat{n}_{\sigma_1}(\mathbf{r}_1) | \alpha \rangle_N \, {}_N\langle \alpha | \hat{n}_{\sigma_2}(\mathbf{r}_2) | 0 \rangle_N}{\hbar\omega - E_\alpha^N + E_0^N + i\eta} \\ &- \sum_\alpha \frac{{}_N\langle 0 | \hat{n}_{\sigma_2}(\mathbf{r}_2) | \alpha \rangle_N \, {}_N\langle \alpha | \hat{n}_{\sigma_1}(\mathbf{r}_1) | 0 \rangle_N}{\hbar\omega - E_0^N + E_\alpha^N - i\eta} \\ &+ 2\pi i \delta(\hbar\omega) n_{\sigma_1}(\mathbf{r}_1) n_{\sigma_2}(\mathbf{r}_2). \end{aligned} \tag{S25}$$

If we take the $\omega \to 0$ limit, the term stemming from the ground state $|0\rangle_N$ in the first two sums cancels the third contribution exactly by the Sokhotski-Plemelj theorem. After taking the limit $\eta \to 0$ the remaining term is purely real and can be written as

$$\begin{aligned} \hat{\chi}_{\sigma_1,\sigma_2}(\mathbf{r}_1, \mathbf{r}_2, \omega = 0) &= 2 \sum_{\alpha \neq 0} \frac{\mathrm{Re}\left[{}_N\langle 0 | \hat{n}_{\sigma_1}(\mathbf{r}_1) | \alpha \rangle_N \, {}_N\langle \alpha | \hat{n}_{\sigma_2}(\mathbf{r}_2) | 0 \rangle_N\right]}{E_0^N - E_\alpha^N} \\ &= 2\mathrm{Re}\left[{}_N\langle 0 | \hat{n}_{\sigma_1}(\mathbf{r}_1) (E_0^N - \hat{H}_+^N)^{-1} \hat{n}_{\sigma_2}(\mathbf{r}_2) | 0 \rangle_N\right], \end{aligned} \tag{S26}$$

where $\hat{H}_+^N$ denotes the Hamiltonian restricted to the excited states. From this, we see that the $\mathbf{R}$ dependence of the screening enters via the $\mathbf{R}$ dependence of the ground state and indirectly via $(E_0^N - \hat{H}_+^N(\mathbf{R}))^{-1}$, i.e. through the excited states. Note that the ground state energy E_0^N does not carry any $\mathbf{R}$-dependence to linear order.

Let us now argue why both contributions are small for the systems under consideration. For a sufficiently gapped system, the change of the ground state along a normal mode and the change of the gap is negligible,

$$\partial_{X^\lambda} |0(\mathbf{R})\rangle_N = \sum_{\alpha \neq 0} \frac{{}_N\langle\alpha|\partial_{X^\lambda}\hat{H}|0\rangle_N}{E_\alpha^N - E_0^N} |\alpha\rangle_N \approx 0, \tag{S27}$$

where $|\alpha\rangle_N$ are the exact N particle excited state, because the denominator in this case dominates. To make this argument more transparent: Typically, the involved energy scales are

$${}_N\langle\alpha|\partial_{X^\lambda}\hat{H}|0\rangle_N \cong 0.01 \text{ eV},$$

while the gap $E_\alpha^N - E_0^N$ is about 2 eV. Thus, because of this scale separation, terms involving the ground state derivative should be negligible. This scale separation is also the reason why the second contribution is negligible. To see this, let us take the derivative of the second contribution along the normal modes. This gives,

$$\partial_{X^\lambda}(E_0^N - \hat{H}_+^N(\mathbf{R}))^{-1} = -(E_0^N - \hat{H}_+^N(\mathbf{R}))^{-1}\partial_{X^\lambda}\hat{H}_+^N(\mathbf{R})(E_0^N - \hat{H}_+^N(\mathbf{R}))^{-1}, \tag{S28}$$

which shows the same scaling as in the ground state case. From this analysis, we conclude that

$$\partial_{X^\lambda}\Xi^{i,\sigma_1;m,\sigma_2}_{n,\sigma_3;j\sigma_4}(\mathbf{R})\,|_{\mathbf{R}^0} \approx 0. \tag{S29}$$

S3 From Quasi-Particles to a Practical Scheme

In the following section, we compile the data underlying the results from the main manuscript, Sec. 3, and provide further supporting information.

S3.1 Technical Details of the Computations

We provide further technical details, how the coupling constants are computed numerically. Our approach is based on the finite difference for the derivative along the normal mode direction. Associated with the normal mode coordinate X^{λ}, there is a polarization vector ξ_{λ} describing the mode pattern, which is given by

$$\xi_{\lambda} = \sum_{s,\alpha} \frac{O_{\lambda}^{s,\alpha}}{\sqrt{M_s}} \mathbf{e}_{s,\alpha}. \tag{S30}$$

This polarization vector is not normalized, but carries units of $\sqrt{\mathrm{mass}}^{-1}$. Its norm is the inverse square root of the mass of the mode λ,

$$M_{\lambda} = ||\xi_{\lambda}||^{-2}. \tag{S31}$$

For convenience we introduce also the normal mode direction $\mathbf{e}_{\lambda}$ of the mode, which is just the normalized polarization vector

$$\mathbf{e}_{\lambda} = \xi_{\lambda}\sqrt{M_{\lambda}}, \tag{S32}$$

which carries no units.

Another comment regarding the implementation of the numerical approach is important. When pursuing a finite difference approach, one has to ensure that the difference of the "right" energies and overlap matrix elements at different geometries as taken, as for example a level crossing can occur, or degenerate levels at the equilibrium geometry split. To circumvent these type of ambiguities in this situation, we introduce the concept of adiabatically connected

eigenvalues and eigenstates. Here we call two normalized, single particle eigenstates $|p_1, \mathbf{e}_\lambda, \delta\rangle$ and $|p_2\rangle$ to be adiabatically connected, if

$$\lim_{\delta\to 0}\Big|\Big|\ |p_1, \mathbf{e}_\lambda, \delta\rangle - |p_2\rangle\ \Big|\Big|^2 = 0 \tag{S33}$$

where $|p_1, \mathbf{e}_\lambda, \delta\rangle$ is the p_1^{th} eigenstate where the relaxed geometry has been displaced in the X^μ's direction $\mathbf{e}_\mu$ by δ. We call two displaced single particle eigenstates $|p_1, \mathbf{e}_{\mu_1}, \delta_1\rangle$ and $|p_2, \mathbf{e}_{\mu_2}, \delta_2\rangle$ adiabatically connected, if they are adiabatically connected to the same state in the relaxed geometry. Correspondingly, their Kohn-Sham eigenvalues, $\epsilon_{p_1}^{(\text{KS})}(\mathbf{R}^0 + \delta_1\mathbf{e}_{\mu_1})$ and $\epsilon_{p_2}^{(\text{KS})}(\mathbf{R}^0 + \delta_2\mathbf{e}_{\mu_2})$, are called adiabatically connected if and only if the corresponding eigenstates are adiabatically connected. We will use these definitions to compute the diagonal and off-diagonal coupling constants,

$$g_i^\lambda = \sqrt{\frac{1}{2\hbar\omega_\lambda^3}}\partial_{X^\lambda}\epsilon_i(\mathbf{R})\Big|_{\mathbf{R}^0}, \tag{S34}$$

and

$$h_{p,q}^\lambda = \sqrt{\frac{1}{2\hbar\omega_\lambda^3}}(\epsilon_q - \epsilon_p)\,\partial_{X^\lambda}\langle p, \mathbf{R}^0 \mid q, \mathbf{R}\rangle\Big|_{\mathbf{R}^0}. \tag{S35}$$

To this end, the atomic configuration $\mathbf{R}^0$ is displaced along the cartesian representation of the normal modes $\{X^\mu\}$ by $\pm\delta$ and compute the eigenvalues $\epsilon_{p_1}^{(\text{KS})}(\mathbf{R}^0 + \delta\mathbf{e}_\mu)$ and $\epsilon_{p_2}^{(\text{KS})}(\mathbf{R}^0 - \delta\mathbf{e}_\mu)$ that are adiabatically connected to $\epsilon_p^{(\text{KS})}(\mathbf{R}_0)$ by checking which states have the maximum overlap with $|p\rangle$ in the displaced geometries. In all the considered cases this procedure gave overlaps with a magnitude over 0.8, and thus a unique identification was possible. From this the partial derivative along the deviation is approximated as

$$\frac{\partial\epsilon_p^{(\text{KS})}(\mathbf{R})}{\partial X^\lambda}\Bigg|_{\mathbf{R}^0} \approx \frac{\epsilon_{p_1}^{(\text{KS})}(\mathbf{R}^0 + \delta\mathbf{e}_\lambda) - \epsilon_{p_2}^{(\text{KS})}(\mathbf{R}^0 - \delta\mathbf{e}_\lambda)}{2\delta\sqrt{M_\lambda}}, \tag{S36}$$

and the analogous identification was used for the off-diagonal coupling constants,

$$\partial_{X^\lambda} \langle p, \mathbf{R}^0 | q, \mathbf{R} \rangle \Big|_{\mathbf{R}^0} \approx \frac{\langle p, \mathbf{R}^0 | q_1, \mathbf{R}^0 + \delta \mathbf{e}_\lambda \rangle - \langle p, \mathbf{R}^0 | q_2, \mathbf{R}^0 - \delta \mathbf{e}_\lambda \rangle}{2\delta \sqrt{M}_\lambda}. \tag{S37}$$

In order to compute the overlaps $\langle p, \mathbf{R}^0 | q_1, \mathbf{R}^0 \pm \delta \mathbf{e}_\lambda \rangle$, we start from the states representation in their respective Gaussian type atom-centered (GTO) basis, as used in CP2K.[3] This takes the form

$$|p, \mathbf{R}^0\rangle = \sum_x a_{x,i} \, |\phi_x\rangle \tag{S38}$$

and

$$|q, \mathbf{R}^0 \pm \delta \mathbf{e}_\lambda\rangle = \sum_y \tilde{a}^{\pm}_{y,k} |\tilde{\phi}^{\pm}_y\rangle, \tag{S39}$$

where x and y are indices for the used GTO basis states $\{|\phi_x\rangle\}$ at the equilibrium geometry and $\{|\tilde{\phi}^{\pm}_y\rangle\}$ at the displaced geometry. Using this we can write

$$\langle p, \mathbf{R}^0 | q_1, \mathbf{R}^0 \pm \delta \mathbf{e}_\lambda \rangle = \sum_{x,y} a_{x,i} T^{\pm}_{x,y} \tilde{a}^{\pm}_{y,k}, \tag{S40}$$

where the transformation matrix $T^{\pm}_{x,y} = \langle \phi_x \mid \tilde{\phi}^{\pm}_y \rangle$ is analytical for GTO basis sets.

S3.2 Functional Dependence of the KS Coupling Constants

To investigate the consistency of the KS coupling constants, as discussed in the main manuscript Sec. 3.1, we considered the pyrazine molecule. This subsection provides the numerical values underlying our analysis in the main manuscript. We also compare the obtained normal modes and coupling constants to the results of Refs.[4,5], which used the Hartree-Fock-based outer-valence Green's function method (OVGF) to compute the coupling constants and MP2 for the mode energies. We used the cc-TZV2P-GTH basis sets from the HFX-basis as implemented in CP2K[3] and employed GTH-pseudopotentials[6,7] for all the calculations.

S3.2.1 Normal Modes

Here, we present the normal mode energies of the ground state of the neutral pyrazine molecule obtained using the different density functionals compared to those obtained by Ref.[4]. All the results are compiled in Table S1.

Table S1: Comparison of the relevant normal mode frequencies $\hbar\omega_\lambda$ of the neutral ground state of pyrazine in [meV].

	mode frequency $\hbar\omega_\lambda$					
method	$\lambda = v_{6a}$	$\lambda = v_1$	$\lambda = v_{9a}$	$\lambda = v_{8a}$	$\lambda = v_2$	$\lambda = v_{10a}$
LDA	72.4	128.5	149.4	196.4	381.4	113.1
PBE	70.9	125.5	150.0	193.3	384.6	113.5
PBE0	73.6	130.8	155.4	203.2	396.5	118.5
BLYP	73.7	124.2	150.2	190.9	383.7	110.1
B3LYP	75.0	128.8	154.5	199.4	393.6	117.7
CAM-B3LYP	74.4	129.7	154.3	200.9	391.4	117.8
MP2[4]	74.0	127.3	156.7	202.5	406.7	113.3

S3.2.2 Kohn-Sham Eigenvalues at the Equilibrium Geometry

In addition to comparing the coupling constants, we present the underlying Kohn-Sham eigenvalues. The underlying data are compiled in Table S2.

Table S2: Comparison of the Kohn-Sham eigenvalues $\epsilon_m^{(\mathrm{KS})}$ for the $m = 0$ and $m = -1$ orbitals for the neutral ground state of pyrazine in [eV].

	KS eigenvalue $\epsilon_m^{(\mathrm{KS})}$	
method	$m = 0$	$m = 1$
LDA	-5.88	-7.37
PBE	-5.86	-7.11
PBE0	-7.32	-8.07
BLYP	-5.82	-6.88
B3LYP	-7.04	-7.74
CAM-B3LYP	-7.64	-8.12

S3.2.3 Diagonal Coupling Constants

This subsection presents the diagonal coupling constants for the pyrazine molecule. We compare the state diagonal coupling constants of the lowest two hole states ($m = 0$ & $m = -1$) between the different functionals used and to the results of Ref.[5] in Table S3. The authors of Ref.[5] have used Koopman's theorem (HF) and the OVGF method to access the coupling constants. Except for one mode, we find very good agreement between the Kohn-Sham approximation and the OVGF method[8].

Table S3: Comparison of the diagonal coupling constants (absolute value) obtained by different methods for pyrazine's two lowest-energy positively charged excited states.

		coupling constant $g_{\lambda,mm}$				
method	state	$\lambda = v_{6a}$	$\lambda = v_1$	$\lambda = v_{9a}$	$\lambda = v_{8a}$	$\lambda = v_2$
LDA	$m = 0$	1.581	0.271	0.008	0.806	0.083
PBE	$m = 0$	1.639	0.273	0.023	0.836	0.091
PBE0	$m = 0$	1.653	0.254	0.000	0.899	0.063
BLYP	$m = 0$	1.512	0.256	0.093	0.840	0.095
B3LYP	$m = 0$	1.557	0.237	0.061	0.887	0.072
CAM-B3LYP	$m = 0$	1.622	0.215	0.036	0.914	0.058
HF[4,5]	$m = 0$	1.842	0.268	0.056	1.159	0.009
OVGF[4,5]	$m = 0$	1.639	0.118	0.031	0.905	0.017
LDA	$m = -1$	0.511	0.436	0.251	0.586	0.040
PBE	$m = -1$	0.539	0.399	0.317	0.581	0.052
PBE0	$m = -1$	0.546	0.463	0.310	0.628	0.055
BLYP	$m = -1$	0.503	0.372	0.370	0.575	0.059
B3LYP	$m = -1$	0.511	0.431	0.351	0.619	0.061
CAM-B3LYP	$m = -1$	0.538	0.474	0.330	0.651	0.061
HF[4,5]	$m = -1$	0.588	0.584	0.461	0.873	0.080
OVGF[4,5]	$m = -1$	0.579	0.529	0.376	0.654	0.009

S3.2.4 Off-Diagonal Coupling Constants

We compare our results to the result of Ref.[5], which used a diabatization method[9,10] employing the third-order algebraic diagrammatic construction (ADC(3))[11–13]. This amounts to computing the full adiabatic potential energy surface along the direction of interest and performing a least-squares fit of the eigenvalues of the diabatic potential energy matrix. As reported

in Table S4, the computationally much cheaper DFT approach gives results close to those obtained using ADC(3).

Table S4: Comparison of the off-diagonal coupling constants (absolute value) obtained by different methods between pyrazine's lowest-energy positively charged excited states.

method	coupling constant $g_{v_{10a},0,-1}$
LDA	1.193
PBE	1.110
PBE0	1.220
BLYP	1.211
B3LYP	1.197
CAM-B3LYP	1.215
ADC(3)[5]	1.403

S3.3 Basis Set Dependence of the KS Coupling Constants

To investigate how sensitive the obtained KS coupling constants are to the chosen basis set size and to provide a convergence check in this regard, we focus on pyrazine. We fix the functional to PBE, employing GTH-pseudopotentials[6,7] and compute the vibrational modes, their energies and the linear coupling constants for the different basis sets. We include DZVP (98), TZ2VP (168), cc-TZ2VP (230) and cc-QZ3VP (440) from the HFX-basis as implemented in CP2K[3], where the numbers in the parentheses are the number of total spherical Gaussian type of orbitals used in the calculation. In Table S5, we compare the normal mode energies computed with the different basis sets. We find only very minor differences, falling in nearly all the cases below 1 meV. This agreement lets us conclude that any of the used basis sets is sufficient to compute the normal modes reliable.

We proceed to the coupling constants. Here we restrict ourselves, as in the case of the functionals, to the energetically lowest hole states. The differences of the diagonal and off-diagonal coupling constants, relative to the values with the cc-QZ3VP basis set, are compiled in Tables S6 and S7, respectively. From this data we conclude that all the different basis sets give consistent coupling constants, with mean absolute errors (relative to cc-QZV3P and

summed over all displayed coupling constants) for the DZVP basis set of 0.021, for TZV2P of 0.0055 and for cc-TZ2VP of 0.0062. The larger MAE for DZVP indicates that this basis set is at the lower end concerning the convergence with respect to the number of basis functions. On the other hand, the other two basis sets (TZV2P and cc-TZV2P) give similar accuracy of a MAE around 0.005. The fact that the MAE is actually not reduced, but slightly increases, by enlarging the basis set gives some confidence that already the TZV2P results are properly converged and that the residual deviations may have another origin. One possible source of deviation is the finite difference approximations, whose expected residual error is of the same order of magnitude, as the one that we find. Altogether, these results indicate that the linear coupling constant are rather insensitive to the used basis set.

Table S5: Comparison of the relevant normal mode frequencies $\hbar\omega_\lambda$ of the neutral ground state of pyrazine in [meV] computed with the PBE functional for different basis sets.

	mode frequency $\hbar\omega_\lambda$					
basis set	$\lambda = v_{6a}$	$\lambda = v_1$	$\lambda = v_{9a}$	$\lambda = v_{8a}$	$\lambda = v_2$	$\lambda = v_{10a}$
DZVP	71.9	126.2	148.8	196.2	386.9	112.6
TZ2VP	71.6	125.5	150.2	193.8	385.8	113.4
cc-TZ2VP	70.9	125.5	150.0	193.3	384.6	113.5
cc-QZ3VP	70.8	125.5	149.9	193.0	383.5	113.5

Table S6: Comparison of the diagonal coupling constants for pyrazine's two lowest-energy positively charged excited states computed with the PBE functional for different basis sets relative to the cc-QZV3P basis set.

		Difference to cc-QZV3P coupling constant $g_{\lambda,mm}$				
basis set	state	$\lambda = v_{6a}$	$\lambda = v_1$	$\lambda = v_{9a}$	$\lambda = v_{8a}$	$\lambda = v_2$
DZVP	$m = 0$	-0.017	0.050	0.007	-0.013	0.000
DZVP	$m = -1$	-0.042	-0.036	-0.052	-0.005	0.002
TZV2P	$m = 0$	0.003	0.009	0.000	0.010	-0.006
TZV2P	$m = -1$	-0.006	-0.012	0.005	0.003	-0.004
cc-TZV2P	$m = 0$	0.011	0.009	0.005	0.011	-0.005
cc-TZV2P	$m = -1$	-0.010	-0.002	-0.003	0.008	-0.003

Table S7: Comparison of the off-diagonal coupling constants for pyrazine's two lowest-energy positively charged excited states computed with the PBE functional for different basis sets relative to the cc-QZV3P basis set.

basis set	Difference to cc-QZV3P coupling constant $g_{v_{10a},0,-1}$
DZVP	-0.011
TZV2P	0.003
cc-TZV2P	-0.001

S3.4 Analytical and Numerical Analysis: QP vs. KS Coupling Constants

Our result from main manuscript Sec. 3.2 that the Kohn-Sham approximation provides reliable estimates of coupling constants for both electron and hole sectors has been exemplified by considering the anthracene molecule. Here, we present the underlying data. For this investigation, we used the CAM-B3LYP functional and the cc-TZV2P-GTH basis set from the HFX-basis for both atom types. Furthermore, GTH pseudopotentials[6,7] have been used.

S3.4.1 Normal Modes

In Table S8, we present the normal mode energies of the neutral anthracene molecule.

Table S8: The normal mode frequencies of the neutral ground state of anthracene in [meV].

λ	v_1	v_2	v_3	v_4	v_5	v_6	v_7	v_8	v_9	v_{10}	v_{11}
$\hbar\omega_\lambda$	8.1	13.5	28.0	28.4	32.3	47.8	48.0	48.5	59.5	60.2	62.8
λ	v_{12}	v_{13}	v_{14}	v_{15}	v_{16}	v_{17}	v_{18}	v_{19}	v_{20}	v_{21}	v_{22}
$\hbar\omega_\lambda$	65.7	73.5	75.8	78.9	81.3	92.4	94.4	94.8	96.1	97.8	101.1
λ	v_{23}	v_{24}	v_{25}	v_{26}	v_{27}	v_{28}	v_{29}	v_{30}	v_{31}	v_{32}	v_{33}
$\hbar\omega_\lambda$	105.1	108.3	113.0	113.6	114.9	115.0	122.7	123.3	125.3	125.4	126.0
λ	v_{34}	v_{35}	v_{36}	v_{37}	v_{38}	v_{39}	v_{40}	v_{41}	v_{42}	v_{43}	v_{44}
$\hbar\omega_\lambda$	126.8	139.3	141.1	145.0	146.4	146.6	149.5	159.1	159.7	159.7	166.1
λ	v_{45}	v_{46}	v_{47}	v_{48}	v_{49}	v_{50}	v_{51}	v_{52}	v_{53}	v_{54}	v_{55}
$\hbar\omega_\lambda$	168.2	175.0	175.6	177.7	183.2	184.3	187.8	196.1	198.7	201.8	207.7
λ	v_{56}	v_{57}	v_{58}	v_{59}	v_{60}	v_{61}	v_{62}	v_{63}	v_{64}	v_{65}	v_{66}
$\hbar\omega_\lambda$	208.0	388.0	388.1	388.8	388.9	389.4	389.6	391.2	391.2	392.8	392.8

S3.4.2 Diabatization Procedure and Off-Diagonal Coupling

As mentioned in the main manuscript, we use a diabatization procedure to benchmark the correction procedure for the off-diagonal coupling constant induced by the mode $\nu = 56$ with energy $\hbar\omega_{56} = 1677cm^{-1}$. This mode choice is motivated by strong off-diagonal hole coupling between the two lowest, energetically well-separated hole states found at the Kohn-Sham level of theory. In addition, this mode is not of A_g symmetry and therefore does not couple diagonally (to linear order), ensuring that the quasi-particle energies remain constant, at least to first order. To obtain an independent benchmark for this off-diagonal coupling constant, we compute the quasi-particle potential energy surface at the G0W0 level of theory. At this level, the potential energy surfaces are computed along the mode coordinate X^ν and plotted in Fig. S1 (a). It exhibits an avoided crossing and, therefore, a hidden conical intersection. Restricting ourselves to these two quasi-hole excitations, we use a diabatization ansatz based on a matrix representation of the Hamiltonian $\hat{H}_\mathrm{eff}$ (in the diabatic basis) of the form

$$\hat{H}_\mathrm{eff} \cong \begin{pmatrix} \epsilon_{-1}^{(\mathrm{QH})}(\mathbf{R}_0) & B(\mathbf{R}) \\ B(\mathbf{R}) & \epsilon_{0}^{(\mathrm{QH})}(\mathbf{R}_0) \end{pmatrix}, \tag{S41}$$

where the function $B(\mathbf{R})$ describes the mixing of these diabatic states at $\mathbf{R}$ by displacing the geometry along X^μ. By demanding that the eigenvalue difference matches the difference in the adiabatic energies at $\mathbf{R}$, one finds for $B(\mathbf{R})$ the expression

$$B(\mathbf{R}) = \mathrm{sgn}(X^\mu)\frac{1}{2}\sqrt{\left[\epsilon_{-1}^{(\mathrm{QP})}(\mathbf{R})) - \epsilon_{0}^{(\mathrm{QP})}(\mathbf{R}))\right]^2 - \left[\epsilon_{-1}^{(\mathrm{QP})}(\mathbf{R}_0)) - \epsilon_{0}^{(\mathrm{QP})}(\mathbf{R}_0))\right]^2}, \tag{S42}$$

where we fixed the phase of $B(\mathbf{R})$ to obtain a differentiable function of X^μ. The obtained $B(\mathbf{R})$ is plotted in Fig. S1 (b). We observe a linear function over the full range, confirming the validity of the ansatz.

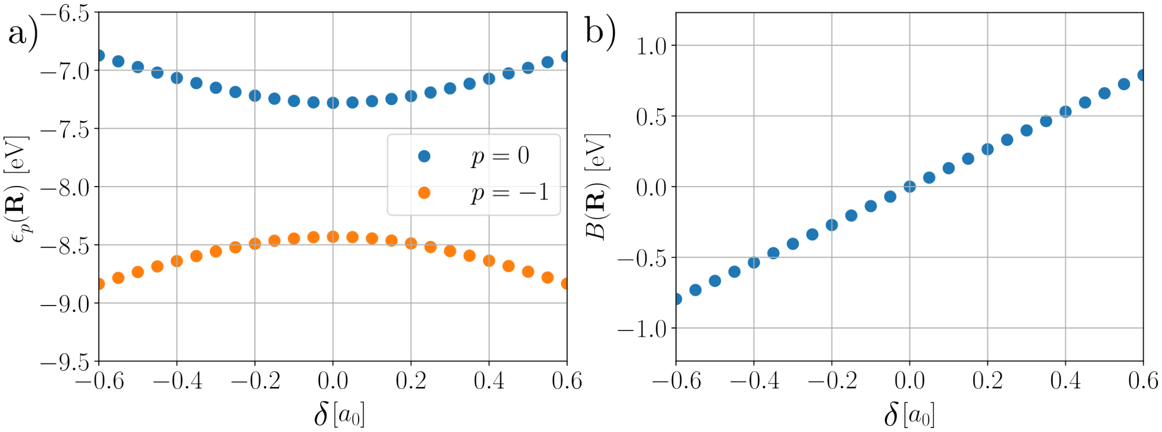

Figure S1: (a) Cut of the quasi-particle potential energy surface along the displacement vector $\mathbf{e}_\mu$ corresponding to mode μ (at G0W0 level) for the first two excited hole states. (b) The plot of the off-diagonal Hamiltonian matrix element $B(\mathbf{R})$ (for definition see main SI text) along the same direction $\mathbf{e}_\mu$ of mode μ.

The coupling constant is obtained by numerical differentiation of $B(\mathbf{R})$ at $\delta = 0$, yielding

$$|g^{(\mathrm{PES})}_{\mu,0-1}| = \sqrt{\frac{1}{2\hbar\omega_\mu^3}}|\partial_{\mathbf{X}^\mu} B(\mathbf{R})| = 0.486. \tag{S43}$$

When comparing this value to the Kohn-Sham result $|g^{(\mathrm{KS})}_{\mu,0-1}| = 0.514$, we find good agreement. Deviations may be traced back to contributions from the quasi-particle shifts of the two states. Thus, we may apply a correction to the Kohn-Sham coupling constants by the multiplication with the factor $(\epsilon^{(\mathrm{G0W0})}_{-1} - \epsilon^{(\mathrm{G0W0})}_{0})/(\epsilon^{(\mathrm{KS})}_{-1} - \epsilon^{(\mathrm{KS})}_{0})$. By this procedure, we arrive at the value $|g^{(\mathrm{corr.})}_{\mu,0-1}| = 0.469$. We conclude that slight deviations exist between the quasi-particle interstate coupling constants from the conical intersection and this correction procedure. However, they seem to be consistent with the expected numerical errors. Like for the diagonal coupling constants, we find good agreement between the KS approximation to the interstate coupling constants and the reference results obtained by the G0W0 method.

S3.5 Extended Numerical Comparison of Coupling Constants

S3.5.1 Comparison to G0W0 and ΔSCF

For the comparison between the KS approximation to the G0W0 and ΔSCF approach as discussed in Sec. 3.3.1 of the main manuscript, we focused on the pentacene molecule. We used the B3LYP[14–16] functional and the TZV2P-GTH basis set from the HFX basis implemented in CP2K[3] for both atom types for this investigation. Furthermore, GTH pseudopotentials[6,7] have been used.

Pentacene: Normal Modes We computed the normal modes of the relaxed, neutral pentacene molecule as described in the main text. From this set of modes we accessed the relevant (totally symmetric) normal modes. The mode energies are tabulated in Table S9.

Table S9: Mode energies of the relevant (totally symmetric) normal modes of the neutral ground state of pentacene in [meV]. These have been used to calculate the coupling constants.

	mode frequency $\hbar\omega_\lambda$						
method	$\lambda = v_1$	$\lambda = v_2$	$\lambda = \lambda = v_3$	$\lambda = v_4$	$\lambda = v_5$	$\lambda = v_6$	$\lambda = v_7$
B3LYP	32.7	76.9	94.1	98.2	125.8	147.4	149.7
	$\lambda = v_8$	$\lambda = v_9$	$\lambda = v_{10}$	$\lambda = v_{11}$	$\lambda = v_{12}$	$\lambda = v_{13}$	$\lambda = v_{14}$
B3LYP	173.9	176.9	185.4	192.4	194.7	392.9	396.1

Pentacene: Diagonal Coupling Constants From the already presented normal modes, we computed the diagonal coupling constants by a finite difference approach with the KS approximation using the B3LYP functional, the G0W0 method on top of the B3LYP calculations, and the ΔSCF calculations as described in the main text. We summarize the obtained diagonal coupling constants in Table S10.

S3.5.2 Comparison to the Outer Valence Green's Function Method

For our comparison to the OVGF method, discussed in Sec. 3.3.2. of the main text, we studied the naphthalene molecule. In this subsection, we compile the underlying data of this

Table S10: Comparison of the diagonal coupling constants of the totally symmetric modes (absolute value) obtained by different methods for the lowest-energy positively and negatively charged excited states of pentacene.

		coupling constant $g_{\lambda,mm}$						
method	state	$\lambda = v_1$	$\lambda = v_2$	$\lambda = v_3$	$\lambda = v_4$	$\lambda = v_5$	$\lambda = v_6$	$\lambda = v_7$
B3LYP	$m = 0$	0.260	-0.015	-0.077	0.081	-0.104	-0.112	0.236
G0W0	$m = 0$	0.219	0.000	-0.024	0.099	-0.111	-0.130	0.300
ΔSCF	$m = 0$	0.196	-0.008	-0.052	0.083	-0.097	-0.115	0.248
		$\lambda = v_8$	$\lambda = v_9$	$\lambda = v_{10}$	$\lambda = v_{11}$	$\lambda = v_{12}$	$\lambda = v_{13}$	$\lambda = v_{14}$
B3LYP	$m = 0$	0.269	0.092	-0.022	-0.302	-0.097	-0.018	-0.022
G0W0	$m = 0$	0.362	0.137	-0.044	-0.319	-0.086	-0.013	-0.017
ΔSCF	$m = 0$	0.282	0.115	-0.033	-0.308	-0.105	-0.013	-0.015
		coupling constant $g_{\lambda,ii}$						
method	state	$\lambda = v_1$	$\lambda = v_2$	$\lambda = v_3$	$\lambda = v_4$	$\lambda = v_5$	$\lambda = v_6$	$\lambda = v_7$
B3LYP	$i = 1$	0.690	-0.219	0.139	-0.111	0.066	0.146	-0.264
G0W0	$i = 1$	0.711	-0.246	0.084	-0.142	0.074	0.159	-0.216
ΔSCF	$i = 1$	0.744	-0.221	0.111	-0.100	0.056	0.148	-0.270
		$\lambda = v_8$	$\lambda = v_9$	$\lambda = v_{10}$	$\lambda = v_{11}$	$\lambda = v_{12}$	$\lambda = v_{13}$	$\lambda = v_{14}$
B3LYP	$i = 1$	-0.318	-0.184	0.053	0.151	0.082	-0.018	-0.016
G0W0	$i = 1$	-0.407	-0.227	0.074	0.159	0.073	-0.020	-0.020
ΔSCF	$i = 1$	-0.322	-0.206	0.060	0.155	0.087	-0.023	-0.024

comparison. We used the CAM-B3LYP functional and the cc-TZV2P-GTH basis set from the HFX basis for both atom types. Furthermore, GTH pseudopotentials have been used.

Naphthalene: Normal Modes The (totally symmetric) normal mode energies of the neutral ground state of the naphthalene molecule and a comparison to the ones obtained by Ref.[17] are given in Table S11. These are relevant for the diagonal coupling constants. For the comparison of the off-diagonal coupling between the OVGF and the KS approximation, the considered modes are compared in Table S12.

Table S11: Comparison of the (totally symmetric) normal mode frequencies of the neutral ground state of naphthalene in [meV]. These have been used to calculate the diagonal coupling constants.

	mode frequency $\hbar\omega_\lambda$						
method	$\lambda = v_1$	$\lambda = v_2$	$\lambda = v_3$	$\lambda = v_4$	$\lambda = v_5$	$\lambda = v_6$	$\lambda = v_7$
CAM-B3LYP	63.8	96.7	129.2	146.7	173.2	184.8	200.9
MP2[17]	63.8	95.6	130.3	145.1	180.8	184.7	201.4

Table S12: Comparison of the normal mode frequencies for the considered interstate coupling modes of the states $m = 0$, $m = -1$, $m = -2$ in [meV]. For details we refer to Ref.[17]. These have been used to calculate the coupling constants.

method	mode frequency $\hbar\omega_\lambda$				
	$\lambda = v_{18}$	$\lambda = v_{22}$	$\lambda = v_{29}$	$\lambda = v_{32}$	$\lambda = v_{34}$
CAM-B3LYP	99.7	203.9	79.0	151.5	191.8
MP2[17]	99.5	202.9	76.6	155.6	193.6
	$\lambda = v_{37}$	$\lambda = v_{39}$	$\lambda = v_{41}$	$\lambda = v_{42}$	
CAM-B3LYP	63.7	144.4	184.3	207.8	
MP2[17]	62.7	143.9	184.0	209.3	

Naphthalene: Diagonal Coupling Constants The respective data of the diagonal coupling of all the totally symmetric modes for KS approximation, the G0W0 and OVGF method are summarized in Table S13.

Table S13: Comparison of the diagonal coupling constants of the totally symmetric modes (absolute value) obtained by different methods for naphthalene's three lowest-energy positively charged excited states.

method	state	coupling constant $g_{\lambda,mm}$						
		$\lambda = v_1$	$\lambda = v_2$	$\lambda = v_3$	$\lambda = v_4$	$\lambda = v_5$	$\lambda = v_6$	$\lambda = v_7$
CAM-B3LYP	$m = 0$	0.119	0.119	0.054	0.181	0.485	0.135	0.509
G0W0	$m = 0$	0.146	0.068	0.038	0.178	0.556	0.156	0.511
OVGF[17]	$m = 0$	0.180	0.025	0.032	0.177	0.329	0.419	0.573
CAM-B3LYP	$m = -1$	0.571	0.559	0.306	0.228	0.060	0.113	0.445
G0W0	$m = -1$	0.541	0.609	0.331	0.223	0.099	0.125	0.427
OVGF[17]	$m = -1$	0.518	0.658	0.320	0.245	0.065	0.158	0.437
CAM-B3LYP	$m = -2$	0.958	0.116	0.019	0.177	0.410	0.169	0.415
G0W0	$m = -2$	0.978	0.189	0.000	0.164	0.251	0.133	0.401
OVGF[17]	$m = -2$	0.986	0.274	0.039	0.169	0.218	0.254	0.420

Naphthalene: Off-Diagonal Coupling Constants The respective data of the off-diagonal coupling constants of the considered modes and states for the KS approximation and OVGF method are summarized in Table S14.

Table S14: Comparison of the off-diagonal coupling constants (absolute value) obtained by the Kohn-Sham approximation for thelowest-energy positively charged states of naphthalene.

method	state	coupling constant $g_{\lambda,mn}$			
		$\lambda = v_{37}$	$\lambda = v_{39}$	$\lambda = v_{41}$	$\lambda = v_{42}$
CAM-B3LYP	$m = 0, n = -1$	0.717	0.228	0.041	0.525
OVGF[17]	$m = 0, n = -1$	0.652	0.211	0.070	0.533
		$\lambda = v_{29}$	$\lambda = v_{32}$	$\lambda = v_{34}$	
CAM-B3LYP	$m = 0, n = -2$	0.623	0.381	0.334	
OVGF[17]	$m = 0, n = -2$	0.534	0.414	0.375	
		$\lambda = v_{18}$	$\lambda = v_{22}$		
CAM-B3LYP	$m = -1, n = -2$	0.441	0.403		
OVGF[17]	$m = -1, n = -2$	0.462	0.420		

S3.6 Exciton Coupling Constants

S3.6.1 Comparison to TD-DFT and XMCQDPT2

Here we provide further information about the calculation of the exciton coupling constants.

Pyrazine: Diagonal Coupling Constants After having parameterized the effective Hamiltonian by employing the KS approximation to the coupling constants using CAM-B3LYP[18], we calculate the singlet excited states using (linear-response)-TD-DFT with the CAM-B3LYP exchange and correlation kernel to access the singlet excited states. The two relevant excited states S_1 and S_2 are at $E_1 = 4.136$ eV and at $E_2 = 5.608$ eV, respectively. Table S15 summarizes their dominant transitions and corresponding exciton amplitudes η. Alternatively, we directly access the diagonal coupling constants by deriving the excited state

Table S15: Dominant transitions and amplitudes for the two relevant singlet states. Only transitions up to $|\eta^{i,m}_{k,\sigma}| > 0.05$ are displayed.

S_1			S_2		
m	i	$\lvert\eta^{i,m}_{1,\sigma}\rvert$	m	i	$\lvert\eta^{i,m}_{2,\sigma}\rvert$
0	1	0.994	-1	1	0.903
-2	8	0.073	-3	2	0.411

energies. We compare our results from the KSη method, which combines the quasi-particle

couplings and the exciton amplitudes (cf. Sec. 3.4.1 of the main manuscript), to the ones obtained in the direct approach from TD-DFT and the XMCQDPT2 method in Table S16. The TD-DFT underlying modes are the same as those in Sec. S3.1.1.

Table S16: Comparison of the diagonal coupling constants (absolute value) obtained by different methods for pyrazine's two lowest-energy charge neutral excited states.

method	state	coupling constant $\mathcal{G}_{\lambda,kk}$		
		$\lambda = v_{6a}$	$\lambda = v_1$	$\lambda = v_{9a}$
KSη	$k = 1$	0.734	0.195	0.538
TDDFT	$k = 1$	0.821	0.161	0.574
XMCQDPT2[19]	$k = 1$	0.779	0.211	0.539
KSη	$k = 2$	1.067	0.970	0.256
TDDFT	$k = 2$	1.114	0.876	0.210
XMCQDPT2[19]	$k = 2$	1.251	1.027	0.213

Pyrazine: Off-Diagonal Coupling Constants We perform the analogous application of the KSη method to compute the off-diagonal coupling constants. We compare these results to the off-diagonal coupling constant from a local diabatization procedure with the energies from TD-DFT and XMCQDPT2. The results are compiled in Table S17.

Table S17: Comparison of the off-diagonal coupling constants (absolute value) obtained by different methods between pyrazine's two lowest-energy charge-neutral excited states.

method	coupling constant $\mathcal{G}_{v_{10a},1,2}$
KSη	1.103
TDDFT	1.234
XMCQDPT2[19]	1.192

References

(1) Bechstedt, F. *Many-Body Approach to Electronic Excitations*; Springer-Verlag Berlin Heidelberg 2015, 2014.

(2) Martin, R. M.; Reining, L.; Ceperley, D. M. *Interacting Electrons - Theory and*

Computational Approaches, hardback ed.; Cambridge University Press: Cambridge, 2016.

(3) Kühne, T. D.; Iannuzzi, M.; Del Ben, M.; Rybkin, V. V.; Seewald, P.; Stein, F.; Laino, T.; Khaliullin, R. Z.; Schütt, O.; Schiffmann, F.; Golze, D.; Wilhelm, J.; Chulkov, S.; Bani-Hashemian, M. H.; Weber, V.; Borštnik, U.; Taillefumier, M.; Jakobovits, A. S.; Lazzaro, A.; Pabst, H.; Müller, T.; Schade, R.; Guidon, M.; Andermatt, S.; Holmberg, N.; Schenter, G. K.; Hehn, A.; Bussy, A.; Belleflamme, F.; Tabacchi, G.; Glöß, A.; Lass, M.; Bethune, I.; Mundy, C. J.; Plessl, C.; Watkins, M.; VandeVondele, J.; Krack, M.; Hutter, J. CP2K: An electronic structure and molecular dynamics software package - Quickstep: Efficient and accurate electronic structure calculations. *The Journal of Chemical Physics* **2020**, *152*, 194103.

(4) Seidner, L.; Stock, G.; Sobolewski, A. L.; Domcke, W. Ab initio characterization of the S1–S2 conical intersection in pyrazine and calculation of spectra. *The Journal of Chemical Physics* **1992**, *96*, 5298–5309.

(5) Seidner, L.; Domcke, W.; von Niessen, W. $\tilde{X}^2A_G{}^-\tilde{A}^2B_{1g}$ conical intersection in the pyrazine cation and its effect on the photoelectron spectrum. *Chemical Physics Letters* **1993**, *205*, 117–122.

(6) Goedecker, S.; Teter, M.; Hutter, J. Separable dual-space Gaussian pseudopotentials. *Phys. Rev. B* **1996**, *54*, 1703–1710.

(7) Hartwigsen, C.; Goedecker, S.; Hutter, J. Relativistic separable dual-space Gaussian pseudopotentials from H to Rn. *Phys. Rev. B* **1998**, *58*, 3641–3662.

(8) von Niessen, W.; Schirmer, J.; Cederbaum, L. S. In *Methods in Computational Molecular Physics*; Diercksen, G. H. F., Wilson, S., Eds.; Springer Netherlands: Dordrecht, 1983; pp 227–248.

(9) Baer, M. Adiabatic and diabatic representations for atom-molecule collisions: Treatment of the collinear arrangement. *Chemical Physics Letters* **1975**, *35*, 112–118.

(10) Mead, C. A.; Truhlar, D. G. Conditions for the definition of a strictly diabatic electronic basis for molecular systems. *The Journal of Chemical Physics* **1982**, *77*, 6090–6098.

(11) Schirmer, J.; Cederbaum, L. S.; Walter, O. New approach to the one-particle Green's function for finite Fermi systems. *Phys. Rev. A* **1983**, *28*, 1237–1259.

(12) Trofimov, A. B.; Stelter, G.; Schirmer, J. A consistent third-order propagator method for electronic excitation. *The Journal of Chemical Physics* **1999**, *111*, 9982–9999.

(13) Harbach, P. H. P.; Wormit, M.; Dreuw, A. The third-order algebraic diagrammatic construction method (ADC(3)) for the polarization propagator for closed-shell molecules: Efficient implementation and benchmarkinga). *The Journal of Chemical Physics* **2014**, *141*, 064113.

(14) Becke, A. D. Density-functional exchange-energy approximation with correct asymptotic behavior. *Phys. Rev. A* **1988**, *38*, 3098–3100.

(15) Vosko, S. H.; Wilk, L.; Nusair, M. Accurate spin-dependent electron liquid correlation energies for local spin density calculations: a critical analysis. *Canadian Journal of Physics* **1980**, *58*, 1200–1211.

(16) Lee, C.; Yang, W.; Parr, R. G. Development of the Colle-Salvetti correlation-energy formula into a functional of the electron density. *Phys. Rev. B* **1988**, *37*, 785–789.

(17) Ghanta, S.; Reddy, V. S.; Mahapatra, S. Theoretical study of electronically excited radical cations of naphthalene and anthracene as archetypal models for astrophysical observations. Part I. Static aspects. *Phys. Chem. Chem. Phys.* **2011**, *13*, 14523–14530.

(18) Yanai, T.; Tew, D. P.; Handy, N. C. A new hybrid exchange–correlation functional using

the Coulomb-attenuating method (CAM-B3LYP). *Chemical Physics Letters* **2004**, *393*, 51–57.

(19) Sala, M.; Saab, M.; Lasorne, B.; Gatti, F.; Guérin, S. Laser control of the radiationless decay in pyrazine using the dynamic Stark effect. *The Journal of Chemical Physics* **2014**, *140*, 194309.
